# Targeting *Acinetobacter baumannii* lipase by coniferous species through metabolomics supported approach

**DOI:** 10.1038/s41598-025-16654-6

**Published:** 2025-09-23

**Authors:** Rania M. Kamal, Ali M. El-Halawany, Mohamed S. Hifnawy, Asmaa M. Otify, Walaa G. Fahmy, Noha M. Elhosseiny, Ahmed S. Attia, Basma M. Eltanany, Laura Pont, Fernando Benavente, Inas Y. Younis, Manal M. Sabry

**Affiliations:** 1 Department of Pharmacognosy, Faculty of Pharmacy, Cairo University, Cairo, 11562 Egypt; 2 Department of Microbiology and Immunology, Faculty of Pharmacy, Cairo University, Cairo, 11562 Egypt; 3 School of Pharmacy, Newgiza University, Giza, 12588 Egypt; 4 Department of Pharmaceutical Analytical Chemistry, Faculty of Pharmacy, Cairo University, Cairo, 11562 Egypt; 5 Department of Chemical Engineering and Analytical Chemistry, Institute for Research on Nutrition and Food Safety (INSA·UB), University of Barcelona, Barcelona, 08028 Spain; 6 Serra Húnter Program, Generalitat de Catalunya, Barcelona, 08007 Spain

**Keywords:** Antivirulence, *Acinetobacter baumannii*, Coniferous, Cupressuflavone, Lipolytic activity inhibition, Drug discovery, Plant sciences, Chemistry

## Abstract

The opportunistic pathogen *Acinetobacter baumannii* is a particularly problematic nosocomial threat worldwide, leading to high morbidity and mortality rates due to its multiple resistance mechanisms, including the production of lipolytic enzymes. Herein, the aerial parts of three coniferous plants, *Pinus canariensis* C. Sm. (PC), *Cupressus lusitanica* Mill. (CL), and *Cupressus arizonica* Greene. (CA), were extracted and fractionated. Among these, the CA extract followed by CL and then PC, exhibited the highest inhibition of bacterial lipase activity, with half-maximal inhibitory concentration (IC_50_) values of 1117 ± 87, 1278 ± 62, and 1926 ± 104 µg/mL, respectively. The methylene chloride fractions of CA and CL extracts exhibited the highest inhibition of bacterial lipase activity, with IC_50_ (940 ± 25 µg/mL and 1103 ± 155 µg/mL), respectively. The metabolite profile of the three extracts, along with their most active fractions, were determined using liquid chromatography-quadrupole-time-of-flight tandem mass spectrometry (LC-QTOF-MS/MS). Interestingly, the metabolite profiles of CL extracts were established here for the first time. A total of 99 secondary metabolites from diverse classes were identified across all samples. Among these, four metabolites were isolated: 3,5-di-*p*-coumaroylquinic acid, epicatechin, cupressuflavone, and rutin. The biflavonoid cupressuflavone showed the lowest IC_50_ value (3812 ± 450 µg/mL). Additionally, partial least squares was applied to assess the key metabolites contributing to the differentiation of the studied bioactivity. Consequently, this study provides novel insights into the bioactivity potential of coniferous plants, demonstrating their value as a natural source of antivirulence agents against the *A. baumannii* lipase enzyme.

**Supplementary Information:**

The online version contains supplementary material available at 10.1038/s41598-025-16654-6.

## Introduction

*Acinetobacter baumannii* (*A. baumannii*) has emerged as a significant challenge for healthcare institutions globally. Currently, it is listed as one of the critical-priority pathogens by the World Health Organization (WHO). Its persistence in hospital settings, coupled with a concerning antibiotic resistance profile, frequently leads to treatment failures, particularly in critically ill patients. As a result, *A. baumannii* has evolved into an outbreak-causing pathogen, marked by a poor prognosis and elevated fatality rates^1^.

*A. baumannii* displays several traits indicative of its adaptation to humans as hosts. One of the reported virulence factors is the presence of lipolytic enzymes. This mechanism involves breaking down host lipids and releasing fatty acids that integrate into the pathogen membranes^2^. Therefore, inhibiting lipolytic activity during the early infection stage may serve as a novel antivirulence strategy combating the *Acinetobacter*-related pathologies associated with hyperlipidemia and obesity. This approach could be implemented through the use of herbal medicines, which are recommended by the World Health Organization (WHO) as a safe and natural alternative to conventional antibiotics^3^.

Conifers are woody plants characterized by needle-like leaves and the production of unisexual cones with bract scales^4^. Coniferous families include Pinaceae, Araucariaceae, Cupressaceae, Podocarpaceae, Cephalotaxaceae, Taxaceae, Phyllocladaceae, and Sciadopityaceae. Conifers encompass approximately 70 genera, such as *Taxus*, *Cupressus*, *Picea*, *Pinus*, *Cedrus*, and *Araucaria*, contributing to over 600 species. Coniferous plants are widely distributed across the Mediterranean region, other areas of the northern hemisphere in Europe, America and East Asia, and subtropical and tropical regions of Central America.

Several researchers have documented the main secondary metabolites found in conifers^5^. These include terpenes (monoterpenes, sesquiterpenes, and diterpenes), nitrogenous compounds (lignans and alkaloids), flavonoids, phenolic acids (benzoic acids and hydroxycinnamic acids), and stilbenes^6^.

Recently, coniferous plants have been reported to exhibit promising antimicrobial activity against various bacterial strains^7^. For instance, the antimicrobial effect of a *Pinus canariensis* ethanolic extract was assessed against different pathogens, including *Enterobacter cloacae*, *Escherichia coli*,* Staphylococcus aureus*, *Enterococcus faecalis*, and three *Candida species (sake*, *albicans*, and *parapsilosis)*. The extract demonstrated minimum inhibitory concentration (MIC) and minimum bactericidal concentration ranging between 0.001 and 10,000 µg/mL and 0.001–1000 µg/mL, respectively^8^. Furthermore, the antibacterial effect of a *Cupressus macrocarpa* diethyl ether extract was evaluated in vitro and in vivo against clinical isolates of methicillin-resistant *S. aureus*, with MIC values ranging from 2 to 8 µg/mL.^9^ This diethyl ether extract not only reduced the growth of all tested isolates by 48.8% but also decreased efflux activity by 29.3%, besides inducing a certain degradation of the cell walls. In a rat model, the mechanism of *C. macrocarpa* extract involved restoring the epidermis, maturing the granulation tissue, and reducing inflammatory cell infiltration. Another recent study explored the antivirulence potential of four coniferous essential oils against *Pseudomonas aeruginosa.* These oils effectively inhibited the expression of soluble virulence factors, such as DNase, lipase, lecithinase, hemolysins, caseinase, and siderophore-like compounds^10^.

The current study aimed to provide a comprehensive metabolite profiling of the methanolic extracts from the aerial parts of three coniferous plants: *Pinus canariensis* C. Sm. (PC), *Cupressus lusitanica* Mill. (CL), and *Cupressus arizonica* Greene. (CA). This profiling was achieved using liquid chromatography-quadrupole-time-of-flight tandem mass spectrometry (LC-QTOF-MS/MS). Today, LC-QTOF-MS/MS is widely used as a highly sensitive and selective tool for the untargeted identification of metabolites in complex samples, such as plant extracts or their fractions^11^. The untargeted metabolomics analysis was further extended through a bioassay-guided approach, applied for the first time to the active fractions of the obtained extracts and their isolated compounds, based on the inhibition of the lipolytic effect of *A. baumannii*. In this context of extensive datasets, chemometrics becomes essential for data interpretation, involving correlation between metabolites and bioactivity. Partial least square (PLS) analysis and variable importance in the projection (VIP) are among the most useful chemometric methods in this scenario^12^. This integrated approach, combining LC-QTOF-MS/MS untargeted metabolomics, bioassays, and chemometrics, revealed metabolites with the greatest potential for antivirulence effects against the troublesome pathogen *A. baumannii*.

## Materials and methods

### Plant material and collection

The aerial parts of PC, CL, and CA were collected from the Orman Botanical Garden (Giza, Egypt) in March 2021. Botanical identification was confirmed by Engineer Therese Labib, consultant in the Orman Botanical Garden and National Gene Bank, Ministry of Agriculture (Cairo, Egypt). Voucher specimens were deposited at the herbarium of the Department of Pharmacognosy, Faculty of Pharmacy, Cairo University, Cairo, Egypt (Codes (3-10-2021Ⅱ), (4-10-2021), and (3-10-2021Ⅲ), respectively).

### Extraction and fractionation

The air-dried aerial parts of the three plants (1 kg each) were individually ground into powder and then extracted with methanol (Me) (EL-Nasr Pharmaceutical Chemicals Company, (Adwic), Cairo, Egypt) by cold maceration at 25 °C (4 × 6 L). The resulting extracts were filtered and evaporated under reduced pressure using a rotary evaporator (Büchi, Switzerland) to obtain dried residues (269 g for PC, 286 g for CL, and 475 g for CA). Different amounts of the dried residues (90 g for PC, 123.6 g for CL, and 290 g for CA) were redissolved in a mixture of water: Me (5:1, v/v). Fractionation using methylene chloride (MC) (ADWIC) was performed (5 × 600 mL) and evaporated, yielding 40 g for PC, 68.6 g for CL, and 85 g for CA. The remaining water fraction underwent chromatographic separation with a Diaion HP-20 column (6 × 44 cm) (Supelco, Bellefonte, PA, USA), resulting in three fractions [100% water (3 L), water: Me (1:1, v/v) (5 L), and 100% Me (5–6 L)]. These three fractions were evaporated, yielding 18, 11.5, and 17.3 g, respectively for PC; 16, 20, and 8 g, respectively for CL; and 16, 42, and 12 g, respectively for CA. All solvents used for extraction and fractionation were analytical grade. Ultrapure water was obtained using Elga PURELAB chorus 1 (with conductivity 0.05 u siemens/cm) (Veolia company, France). Extracts and fractions were stored at 4 °C before LC-QTOF-MS/MS and biological analyses.

### Investigation of the antibacterial activity and antivirulence effect

#### Bacterial strain and growth conditions

The test strain was the multi-drug resistant *A. baumannii* strain (AB5075)^13^. It was routinely grown aerobically at 37 °C in Luria Bertani (LB) broth (Becton Dickinson, Franklin Lakes, NJ, USA) with constant shaking at 180 rpm, or on LB agar plates.

##### Tetrazolium/formazan assay

The broth microdilution method^14^ was used to determine the MIC of PC, CL, and CA extracts, their fractions, and orlistat, a known pancreatic lipase inhibitor recently described as a potential bacterial lipases inhibitor served as the positive control^15^. Initially, a single isolated colony of AB5075’s LB agar plate was used to inoculate 3 mL of LB broth, which was then shaken overnight at 37 °C and 180 rpm. Subsequently, the culture was 100-fold diluted in fresh LB broth and was grown under the same conditions until reaching an optical density at 600 nm (OD_600_) of 0.6 arbitrary units, measured using a Synergy 2 microplate spectrophotometer (BioTek, Winooski, VT, USA). A four mL sample of the obtained culture was centrifuged at 6000 rpm for 5 min, washed twice, and then suspended in sterile normal saline (Otsuka Pharmaceuticals, Cairo, Egypt) to meet 0.5 McFarland’s turbidity standard (OD_600_ = 0.08–0.1 arbitrary units). This suspension was then diluted 20-fold in sterile Mueller-Hinton broth (MHB) (Oxoid, Hampshire, UK) to serve as the bacterial inoculum. In U-bottomed microtiter plates, 15 µL of the inoculum suspension was combined with equal volumes of sterile, double-concentrated MHB and two-fold serial dilutions of each tested sample in normal saline, resulting in final concentrations ranging from 512 to 0.25 µg/mL. The plates were then incubated stationary at 37 °C for 24 h, and the MIC value was determined as the lowest concentration of the tested extract/Fra. that showed no visible growth.

The method described by M. Radaelli et al.^16^ was employed with minor modifications to overcome the challenge of measuring turbidity as an indicator of bacterial growth in samples that exhibited intense coloration or precipitation tendencies. Briefly, the test was conducted using 96-well U-bottom microplates with an assay volume of 165 µL per well. Each well was filled with 150 µL of MHB containing a concentration range of 420–640 µg/mL for all samples. Furthermore, 15 µL of MHB bacterial culture, prepared as mentioned above, was inoculated into each well. Subsequently, the microplates were incubated for 24 h at 37 °C. After this incubation period, 16.5 µL of a 0.5% aqueous solution of 2,3,5-triphenyl tetrazolium chloride (TTC) (Sigma-Aldrich, St. Louis, MO, USA) was added to all wells. The plates were re-incubated at 37 °C for 2 h. The redox indicator (TTC) was used to differentiate metabolically active cells from inactive ones. In wells demonstrating positive bacterial growth, the colorless TTC was reduced to pink/red 1,3,5-triphenyl formazan (TPF). Both positive (excluding the tested substance) and negative (excluding the test microorganism) controls were included in the assay, and the experiments were conducted in triplicate. The effective inhibitory concentration (EIC) can be defined as the lowest concentration preventing the red color formation.

##### Growth curve assay

To assess the effect of the extracts and their fractions on the growth kinetics of *A. baumannii*, growth curves were constructed as previously reported with some modifications^17^. The OD_600_ of the overnight culture of AB5075, grown for 18–20 h, was adjusted at 0.5 arbitrary units. This culture was then diluted 1:100 in fresh LB broth. The diluted culture was individually mixed with the studied samples to achieve a final concentration below the minimum inhibitory concentration (sub-MIC) as follows: 256 µg/mL for PC extract, 128 µg/mL for CL and CA extracts and also for MC fraction of CA extract (Fra. MC. CA), 64 µg/mL for MC fractions of PC and CL extracts (Fra. MC. PC and Fra. MC. CL, respectively), and 512 µg/mL for all other fractions. Dimethyl sulfoxide (DMSO) (Sigma-Aldrich) was used as a negative control, while orlistat served as a positive control, tested at final concentrations of 64 µg/mL and 128 µg/mL. Cultures were incubated at 37 °C with shaking at 180 rpm. Aliquots were collected from each culture every hour for 10 h and then 24 h post-inoculation. The growth curves were generated by plotting the measured OD_600_ values against time in hours.

##### Lipase assay

Lipase activity was measured through a colorimetric assay using *p*-nitrophenyl palmitate (*p*NPP) (Sigma-Aldrich) as a substrate, as described previously with some modifications^18^. Briefly, a 100 µL reaction mixture consisting of equal volumes of a reaction buffer (2 mM *p*NPP, 50 mM Tris-HCl (pH = 7.2), and 2% acetonitrile) and bacterial culture (OD_600_ = 0.6 arbitrary units) containing the tested samples (extract/Fra.) was prepared. The tested concentrations were non-growth inhibitory ones to avoid interference of growth inhibition on the lipolytic activity. This reaction mixture was added to a flat-bottomed, clear 96-well plate and incubated at 37 °C. The development of the yellow *p*-nitrophenol (*p*NP) resulting from *p*NPP hydrolysis was monitored at 410 nm at zero time and 3 h post-incubation, against a negative control (absence of tested sample). The the lipolytic inhibition percentage was calculated according to the following equations:

%ΔA (control or test) = (A _3 h_/A _0 h_) X 100, where %ΔA is the percentage change in absorbance.

Remaining lipolytic activity (%) = (%ΔA _test_/%ΔA _control_) X 100.

Lipolytic inhibition (%) = 100 - remaining lipolytic activity.

In addition, the concentration required to achieve 50% lipase inhibition (half-maximal inhibitory concentration, IC_50_) was determined for each tested sample. This assessment was conducted as described above, employing a final concentration range of 16–2048 µg/mL for all the tested samples^19^. The IC_50_ values were determined through four-parameter logistic regression from at least three independent experiments using GraphPad Prism software (version 9.0).

##### Statistical analysis for biological activity assays

All assays were conducted in triplicate. Statistical analysis was performed using the GraphPad Prism program (version 9.0, USA), applying one-way analysis of variance (ANOVA), or Microsoft Excel (2016), applying unpaired Student’s *t*-test. All results were expressed as the mean ± the mean standard error (SEM). *P* values less than 0.05 were considered statistically significant.

###  LC-QTOF-MS/MS metabolite profiling

All the chemicals used were of liquid chromatography-mass spectrometry (LC-MS) grade (Merck, Darmstadt, Germany). Samples were prepared by dissolving 10 mg of each extract/Fra. in 1 mL of methanol, followed by centrifugation at 13,000 g, and filtration through 0.22 m nylon syringe filters. LC-QTOF-MS/MS experiments were carried out in triplicate on a 1260 Infinity liquid chromatograph coupled to a 6546 LC/QTOF mass spectrometer with an orthogonal electrospray ionization (ESI) interface (Agilent Technologies, Waldbronn, Germany). A Zorbax SB-C18 column (150 mm total length, 2.1 mm internal diameter, 5 μm particle size, 90 Å pore diameter, Agilent Technologies) was employed for separation of samples (5µL) under gradient elution at 350 µL/min, using water and acetonitrile mobile phases (both with 0.1% (v/v) of formic acid). The elution gradient and the QTOF mass spectrometer parameters in positive and negative ESI modes was as described in our previous work^20^. LC-QTOF-MS/MS raw data was visualized using MassHunter software (B.06.00 Service Pack1, Agilent Technologies). The raw data were converted to mzXML format using the open-source software MSConvert 3.0 (https://www.proteowizard.org). Subsequently, the mzXML files were imported into the data mining open-source software MZmine 2.53 (https://github.com/mzmine/mzmine2/releases/tag/v2.53) for peak picking, deconvolution, deisotoping, alignment, and formula prediction. Metabolites were tentatively identified based on peak retention time (R_t_), molecular formula, characteristic MS/MS fragmentation pattern in both the negative and positive ESI modes, and comparison with previously reported literature data and online databases (e.g. PubChem, https://pubchem.ncbi.nlm.nih.gov/, Reaxys chemical database, https://www.reaxys.com, MassBank of North America, https://mona.fiehnlab.ucdavis.edu/). As a result, the annotated metabolites were identified at a high confidence level (Level 2: probable structure by MS, MS/MS experimental data, and library/bibliography MS/MS)^20^.

### Isolation of metabolites and characterization by NMR

Based on the biological activity results, the fraction yields, or thin layer chromatography (TLC) analyses, Fra. MC. CA and water: Me (1:1, v/v) fraction of CL extract (Fra. 50%Me. CL) were selected for further chromatographic isolation of their main bioactive compounds (Supplementary Fig. S1 online). TLC plates (Merck, 250 μm thickness and KGF RP-18 Silica gel 60) were used for TLC analyses, visualizing the spots under UV-visible light at 254 and 365 nm. *p*-Anisaldehyde/H_2_SO_4_, followed by heating at 110 °C, and aluminum chloride were used as spraying reagents.

All solvents used in column chromatography were of analytical grade (ADWIC, Cairo, Egypt). Fra. 50%Me. CL (15 g dissolved in the minimum amount of distilled water) was fractionated in a polyamide stationary phase column (Fluka, Darmstadt, Germany, 7 × 15 cm) using a gradient elution with water: Me mixtures, at 10% increments, to yield eleven fractions (Fra. 1- Fra. 11). Based on TLC analyses, fractions with similar profiles were pooled to give 4 subfractions (Sub-Fra. A, B, C, and D). Sub-Fra. C was separated in a Sephadex LH-20 column (Sigma-Alcrich, St. Louis, MO, USA, 2.5 × 30 cm) using water : Me (1:1, v/v), to yield two subfractions (Sub-Fra. 1 C and 2 C). Sub-Fra. 1 C and 2 C (100 and 50 mg, respectively) were applied to silica gel columns (Merck, 1.5 × 30 cm and 1 × 15 cm, respectively) and gradiently eluted with MC: Me mixtures, at 1% increments, to yield compounds C**1** and C**2** (71 mg and 40 mg, respectively). Sub-Fra. D (50 mg) was separated in another Sephadex LH-20 column (2.5 × 30 cm) using water : Me (1:1, v/v), to yield compound C**3** (30 mg).

Fra. MC. CA (20 g) was separated in another silica gel column (4 × 25 cm) starting from 100% hexane and increasing polarity (with 10% increment until 90%) with MC, followed by 100% ethyl acetate. Several Sub-Fra. were collected and, after TLC analyses, those with similar profiles were pooled to give 4 derived Sub-Fra (Sub-Fra. E, F, G, and H).

Sub-Fra. G (100 mg) was separated in another silica gel column (1.5 × 28 cm) starting from 100% MC until 85:15 MC: Me with 5% Me increments, leading to the isolation of compound C**4** (84 mg).

The NMR experiments for all the isolated compounds were performed using a BrukerHigh-Performance Digital FT-NMR spectrometer Avance III 400 MHz (Bruker, Corp., Billerica, MA, USA) at the Faculty of Pharmacy, Nuclear Magnetic Resonance laboratory, Cairo University, Cairo, Egypt. Deuterated dimethyl sulfoxide (DMSO-d_6_) or Deuterated methanol (CD_3_OD)] were used as solvents and tetramethyl silane as internal standard to record the NMR spectra.(-)-Epicatechin (≥ 97.0%) and rutin (≥ 94.0%) analytical standards ≥ 94.0% were obtained from Sigma Aldrich. No analytical standards were available for the rest of isolated compounds.

### Multivariate data analysis

The compound features from the MZmine output were converted into individual CSV files for each sample (extracts of PC, CL, and CA, along with MC fractions of CL and CA, all analyzed in triplicate). A feature ID number, pseudomolecular ion (*m/z*), retention time and peak intensity for each feature in all samples were recorded {15 columns (sample extracts and fractions) × 99 rows (peak intensity for each identified compound)}. Then, the data set was mean-centered, exported to SIMCA-P (version 14, Umetrics, Umeå, Sweden), and Pareto scaled before multivariate data analysis, together with the vector containing the lipolytic activity inhibition.

PLS analysis was used to uncover the correlation between the bioactivity and the LC-MS dataset of identified metabolites. Subsequently, the variable importance in the projection (VIP) values from the PLS analysis were utilized to pinpoint the metabolites that have the strongest influence on bioactivity (with a VIP value ≥ 0.7). The PLS was validated through permutation tests (*n* = 200), regression analysis, and assessment of model fitness parameters (goodness of fit: R2Y; goodness of prediction: Q2Y, and cross-validation: CV-ANOVA, *p* < 0.01).

## Results and discussion

### Investigation of the antibacterial activity and antivirulence effect

#### Tetrazolium/formazan test

The standard MIC procedure did not allow an accurate determination of the MIC value due to interference from the dark background color of the tested extracts and fractions. Instead, determination of the EIC using the TTC assay was used. Table 1 presents MIC and EIC values for all tested samples. Notably, Fra. 50% Me. CA showed the highest inhibitory activity, with the lowest EIC recorded at 500 µg/mL. In contrast, the majority of the remaining samples exhibited elevated EIC values of 620 µg/mL or higher, with a few falling between 500 and 600 µg/mL, such as Fra. 50% Me. CL and CA extract. In summary, all tested extracts, fractions, and orlistat demonstrated minimal direct antimicrobial activity against *A. baumannii*.

**Table 1:** MIC and EIC values against *A. baumannii* lipolytic activity for the methanolic extracts of the three coniferous plants and their fractions.

**Sample**	**MIC (µg/mL)**	**EIC (µg/mL)**
*Pinus canariensis* ** C. Sm. (PC)**		
PC	> 256	640
Fra. MC. PC	> 64	600
Fra. 50% Me. PC	> 512	> 640
Fra. 100% Me. PC	> 512	> 640
*Cupressus lusitanica * **Mill. (CL)**		
CL	> 128	> 640
Fra. MC. CL	> 64	> 640
Fra. 50% Me. CL	> 512	580
Fra. 100% Me. CL	> 512	620
*Cupressus arizonica * **Greene (CA)**		
CA	> 128	560
Fra. MC. CA	> 128	620
Fra. 50% Me. CA	> 512	500
Fra. 100% Me. CA	> 512	> 640
**Control**		
Orlistat	> 128	640

MIC: minimum inhibitory concentration, EIC: effective inhibitory concentration, Fra.: fraction, MC: methylene chloride, Me: methanol.

##### Growth curve assay

**Fig. 1:**
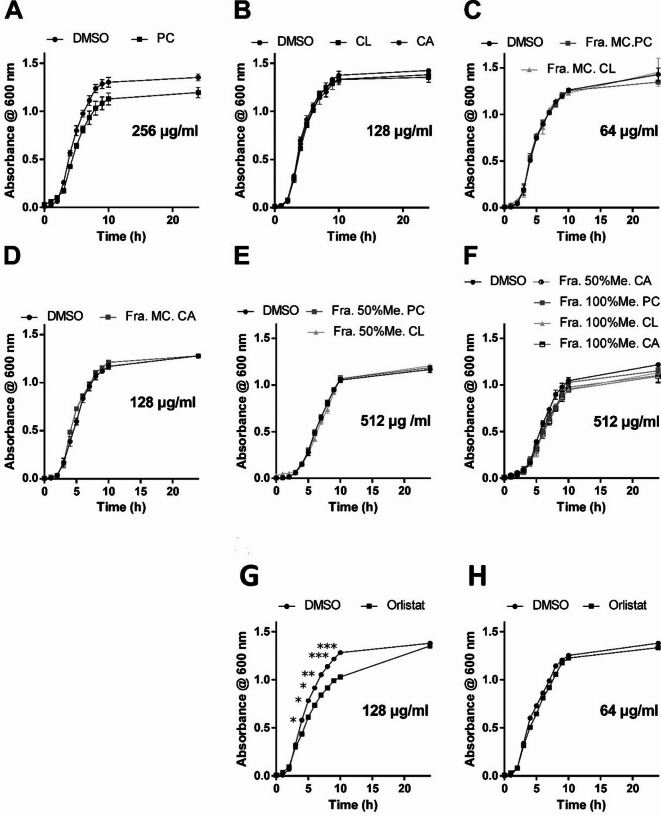
Effect of orlistat, PC, CL, CA extracts and their fractions on the growth pattern of *A. baumannii*, using DMSO as control. The data presented is the average of three independent experiments and the error bars are the standard error of the mean (SEM). The difference between the control and each tested sample at each time point was considered statistically significant as determined by the student’s *t*-test, considering *p < 0.05, **p < 0.01, and ***p < 0.001.

A growth curve assay was conducted to assess the impact of PC, CL, or CA extracts and their fractions at sub-MIC concentrations for any growth defect of *A. baumannii*. No differences were observed in the growth patterns when exposed to either extracts, fractions, or the control (DMSO) during the first 10 h or even after 24 h of incubation (Fig. 1A-F). In contrast, the positive control, orlistat, demonstrated a significant reduction in growth extent during the mid-logarithmic and early stationary phases at a concentration as low as 128 µg/mL, but not 64 µg/mL, when compared to DMSO. (Fig. 1G& H).

##### Lipase assay

**Fig. 2:**
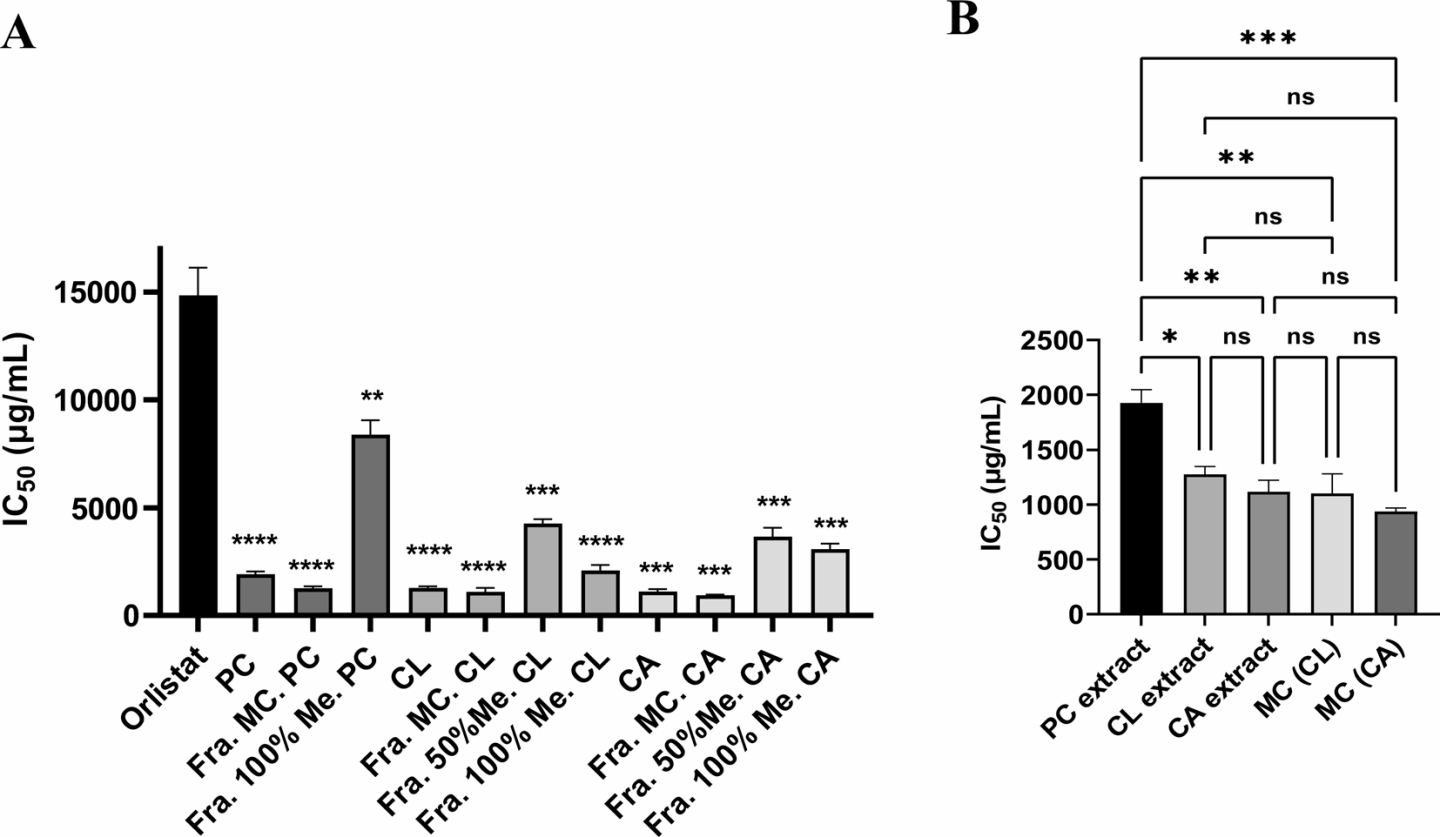
Effect of PC, CL, CA extracts and their fractions on the *A. baumannii* lipolytic activity. (**A**) IC_50_ values of the extracts and their fractions were all compared to orlistat by the student’s *t*-test. (**B**) IC_50_ values of the selected extracts and the most active fractions were compared to each other by one-way ANOVA followed by Tukey’s multiple comparison test. The data presented are the average of at least three independent experiments and the error bars are the SEM. In all cases, values were considered significantly different considering *p < 0.05, **p < 0.01, ***p < 0.001, ****p < 0.0001, and ns = non-significant.

The lipase assay was used to examine the effect of PC, CL, and CA extracts, along with their fractions, on the lipolytic activity of *A. baumannii*, and hence IC_50_ values were determined (Fig. 2A). The three extracts exhibited good lipase inhibition activities with IC_50_ values of 1926 ± 104, 1278 ± 62, and 1117 ± 87 µg/mL for PC, CL, and CA, respectively. These values were significantly lower than the IC_50_ value of the positive control, orlistat, (14835 ± 920 µg/mL) (Table 2). Among the extracts, CA extract demonstrated the highest activity, and among its fractions, Fra. MC. CA exhibited the most significant activity with an IC_50_ value of 940 ± 25 µg/mL. The second most potent fraction among all tested ones was Fra. MC. CL with an IC_50_ value of 1103 ± 155 µg/mL. Consequently, these two fractions were selected with the extracts for LC-QTOF-MS/MS metabolite profiling.

**Table 2:** IC_50_ values against *A. baumannii* lipolytic activity for the methanolic extracts of the three coniferous plants and their fractions.

Sample	IC_50_ (µg/mL) (Mean ± SEM)
*Pinus canariensis* ** C. Sm. (PC)**	
PC	1926 ± 104
Fra. MC. PC	1274 ± 63
Fra. 50% Me. PC	350,504 ± 33,888
Fra. 100% Me. PC	8390 ± 565
*Cupressus lusitanica * **Mill. (CL)**	
CL	1278 ± 62
Fra. MC. CL	1103 ± 155
Fra. 50% Me. CL	4265 ± 177
Fra. 100% Me. CL	2096 ± 220
*Cupressus arizonica * **Greene (CA)**	
CA	1117 ± 87
Fra. MC. CA	940 ± 25
Fra. 50% Me. CA	3656 ± 355
Fra. 100% Me. CA	3084 ± 204
**Control**	
Orlistat	14,835 ± 920

IC_50_: half-maximal inhibitory concentration, Fra.: fraction, MC: methylene chloride, Me: methanol.

Having a close look at the comparison between the tested extracts and the selected fractions (MC. CL and MC. CA) (Fig. 2B), it appeared that both the CL and CA extracts were significantly more potent (lower IC_50_) than the PC extract. Also, the two fractions Fra. MC. CL and Fra. MC. CA had IC_50_ values which were significantly lower than that of the PC extract. On the other hand, the differences among the two extracts CL and CA and the two fractions MC. CL and MC. CA were non-significant.

It is worth mentioning that the lipase inhibitory effects of extracts of six commonly consumed *Brassica* plants were tested before, among them, Curly Kale, which showed the greatest inhibition by 26% at 256 µg/mL concentration^11^.

###  LC-QTOF-MS/MS metabolite profiling

**Fig. 3:**
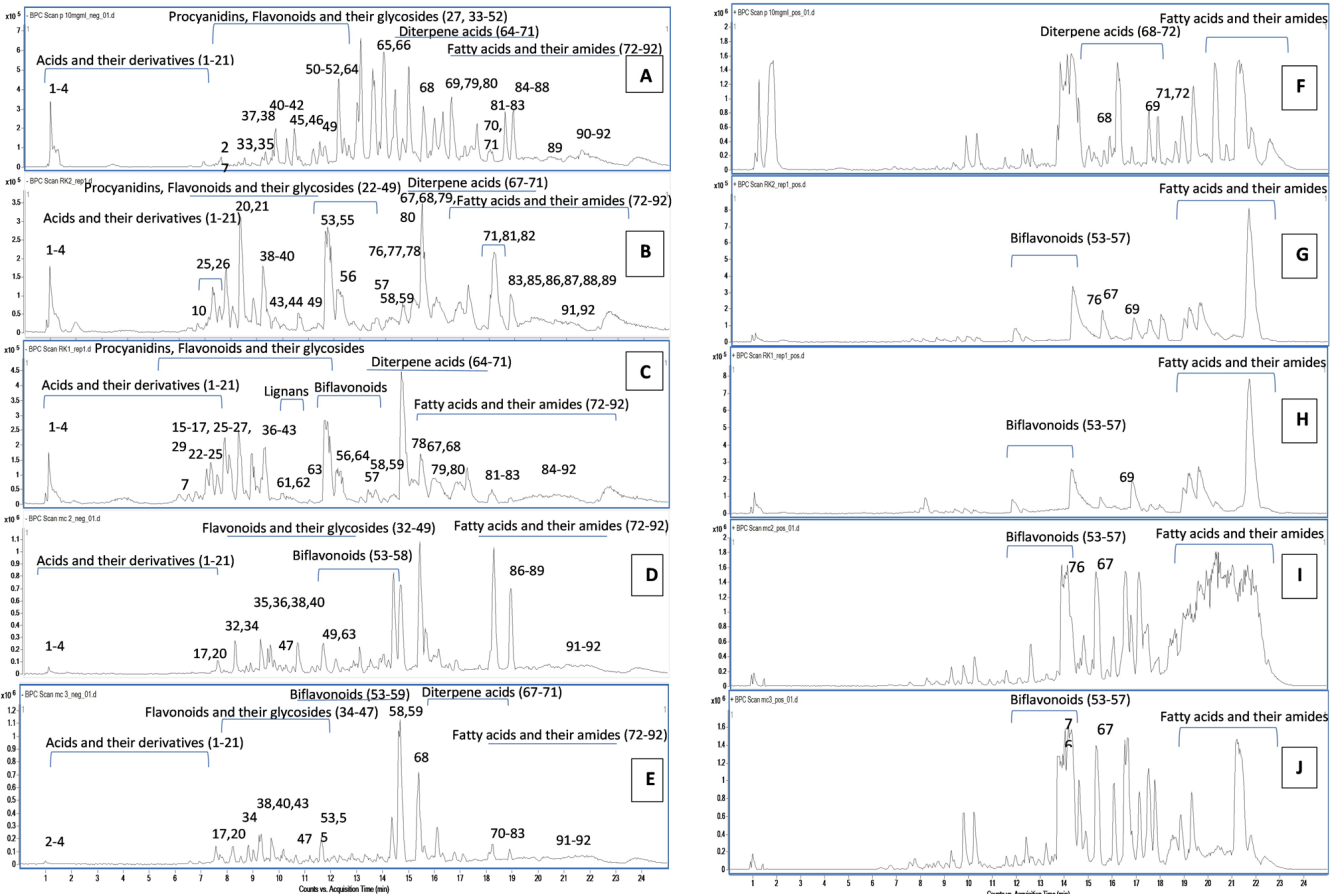
LC-QTOF-MS/MS base peak chromatograms of the PC, CA, and CL extracts and their most active fractions in negative ESI mode: **(A)** PC extract, **(B)** CL extract, **(C)** CA extract, **(D)** Fra. MC. CL, **(E)** Fra. MC. CA, and in positive ESI ionization mode: **(F)** PC extract, **(G)** CA extract, **(H)** CL extract, **(I)** Fra. MC. CA, **(J)** Fra. MC. CL. The peak numbers of the identified metabolites are as listed in Table 3.

PC, CL, and CA extracts and their most active fractions (Fra. MC. CL and Fra. MC. CA) were analyzed by LC-QTOF-MS/MS, Table 3 summarizes the identified metabolites, including the experimental *m/z* of the detected pseudomolecular ions in negative and positive ESI modes, MS/MS fragments, R_t_, molecular formula, and chemical classes. The base peak chromatograms of the metabolite profiles are displayed in Fig. 3. A total of 99 metabolites were annotated, with 69 metabolites in the negative ESI mode, 9 in the positive ESI mode, and 21 in both modes. The annotated metabolites, totaling 61 in PC and CL, 63 in CA, 43 in Fra. MC. CL, and 45 in Fra. MC. CA, belonged to various classes, including 21 organic and phenolic acids, 6 catechins and their derivatives, 25 flavonoids and their glycosides, 7 biflavonoids, 4 lignans, 8 diterpenes, 21 fatty acids and their amides, and 7 miscellaneous. This represents the first comprehensive metabolite profiling involving CL extract, in comparison to CA and PC extracts, as well as specific biologically relevant fractions (Fra. MC. CL and Fra. MC. CA). In both negative and positive ESI modes, detection of biflavonoids was demonstrated with high abundance in CL, CA extracts and their fractions in contrast to the PC extract. On the other hand, fatty acids (in negative ESI mode) and their amides (in positive ESI mode) showed high abundance in all the samples.

**Table 3:** Identified metabolites in three coniferous plants’ methanolic extracts and their fractions using LC-QTOF-MS/MS in negative and positive ESI ionization modes.

Peak no.	Rt (min)	Pseudomolecular ion [M–H]^–^(m/z)	MS/MS fragments (m/z) Negative mode	Pseudomolecular ion [M + H]^+^ (m/z)	MS/MS fragments (m/z) Positive mode	Molecular Formula	Error (ppm)	Name	Extract/Fraction					Reference
									PC	CL	Fra. MC. CL	CA	Fra. MC. CA	
Acids and their derivatives (organic and phenolic)														
1	1.127	533.1718	191*			C_19_H_34_O_17_	0.98	Quinic acid derivative	+	+	+	+	-	^25^
2	1.13	191.0552	173, 155, 111, 93, 85*			C_7_H_12_O_6_	1.1	Quinic acid	+	+	+	+	+	^27^
3	1.785	169.0137	125*			C_7_H_6_O_5_	3.22	Gallic acid	+	+	+	-	+	^25^
4	1.427	173.0434	**173**,** 155**, 93*, 67			C_7_H_10_O_5_	6.02	Shikimic acid	+	+	+	+	+	^27^
5	3.982	482.745	337*, 319, **163**			C_25_H_22_O_10_	1.1	3,5-di-*p*-coumaroyl quinic acid	+	+	-	-	-	^28^
6	5.813	175.0607	115, 85,79*			C_7_H_12_O_5_	2.82	Unknown organic acid	+	-	-	-	-	-
7	5.845	153.0182	135, 109* 81	155.0336	137, 111	C_7_H_6_O_4_	7.35	Protocatechuic acid	-	-	-	+	-	^60^
8	5.86	168.3324	152, 123, 108			C_8_H_8_O_4_	7.04	Homogentisic acid	+	-	-	-	-	^61^
9	6.065	151.039	142, 131			C_8_H_8_O_3_	7.02	Vanillin (phenolic aldehyde)	+	-	-	-	-	^60^
10	6.066	329.0869	167*, 152			C_14_H_18_O_9_	2.74	Dihydroxybenzoic acid methyl ether-*O*-hexoside	-	+	-	-	-	^25^
11	6.265	167.0341	152*, 108			C_8_H_8_O_4_	5.25	5-Methoxysalicylic acid	-	+	+	-	-	^47^
12	6.42	315.0707	153*,** 109**			C_13_H_16_O_9_	3.34	Protocatechuic acid-hexoside	-	+	-	+	-	^60^
13	6.622	183.0282	168, 124*			C_8_H_8_O_5_	4.33	Methyl gallate	+	-	-	-	+	^62^
14	6.916	341.0871	**179**, 135*			C_15_H_18_O_9_	2.06	Caffeoyl-*O*-hexoside	+	-	-	-	-	^62^
15	7.08	443.1905	**281**,119, 113, 101, 71, 59*,			C_21_H_32_O_10_	3.54	Dihydrophaseic acid 4′-*O*-*β*-d-glucopyranoside	-	+	-	+	-	^63^
16	7.253	337.0915	**191**, 163, 119*			C_16_H_18_O_8_	4.11	Coumaroyl quinic acid	-	+	-	+	-	^27^
17	7.477	163.0393	119*	165.0544	121*	C_9_H_8_O_3_	4.68	*p*-coumaric acid	+	+	+	+	+	^9^
18	7.493	179.0347	135*	181.085	137*	C_9_H_8_O_4_	1.57	Caffeic acid	+	-	-	-	-	^62^
19	7.53	163.0391	119*, 93	165.0544	121*	C_9_H_8_O_3_	5.9	*Trans*−2-hydroxy cinnamic acid	+	-	-	-	-	^64^
20	7.83	193.0494	134,117			C_10_H_10_O_4_	6.35	*Trans-*ferulic acid	+	+	+	+	+	^65^
21	8.188	399.0916	**191**			C_17_H_20_O_11_	4.21	Unknown quinic acid derivative	-	+	-	+	-	-
Catechins and their derivatives														
22	6.07	305.07	219, 261,219, 165, 139, 137, 125*, 109			C_15_H_14_O_7_	4.17	Epi/gallocatechin	-	+	-	+	-	^66^
23	6.40	593.1278	407,177*,** 125**	595.1461	409, 179*,** 127**	C_30_H_26_O_13_	−2.89	Epigallocatechin-epicatechin	-	-	-	+	-	^66^
24	6.509	451.1221	289*, 245			C_21_H_24_O_11_	5.5	Catechin-7-*O*-*β*-D-glucopyranoside	-	+	-	+	-	^66^
25	7.19	577.1334	407, **289**, 245,203, 161, 125*	579.1527	**291**, 127*	C_30_H_26_O_12_	3.37	Procyanidin B	-	+	-	+	-	^9^
26	7.20	305.0653	261, 219, 203, 179,167, 125*			C_15_H_14_O_7_	4.5	Gallocatechin	-	+	-	+	-	^66^
27	7.54	289.0707	289, 271, **245**, 179, 159, 151, 137, 109*	291.0874	291, 139	C_15_H_14_O_6_	3.66	Epi/Catechin	+	+	-	+	-	^9^
Flavonoids and their glycosides														
28	6.526	451.1221	289* 245, 165			C_21_H_24_O_11_	5.5	3-Hydroxyphloretin 2’-*O*-glucoside	-	+		+	-	^67^
29	7.58	289.0703	271,109*			C_15_H_14_O_6_	5.04	Pentahydroxyflavan	-	-	-	+	-	^47^
30	8.073	593.1489	446, 285*, 255, 151	595.1678	287*, 271	C_27_H_30_O_15_	3.89	Kaempferol rhamnosyl-hexoside	-	+	-	-	-	^68^
31	8.134	449.1078	**287**, 269*			C_21_H_22_O_11_	2.52	Eriodictyol-7-*O*-glucoside	-	+	-	-	-	^9^
32	8.737	609.1437	301, 300*, 151	611.1615	303*,** 302**	C_27_H_30_O_16_	3.95	Rutin	-	+	+	+	-	^33^
33	8.784	465.101	303*, 285			C_21_H_22_O_12_	6.11	Taxifolin-*O*-hexoside	+	-	-	-	-	^35^
34	8.799	433.1699	271*, 151, 119			C_21_H_22_O_10_	−3.41	Naringenin-7-*O*-hexoside	-	+	+	-	+	^69^
35	8.922	463.0856	316*, 315, **301**,** 300**			C_21_H_20_O_12_	5.6	Myricetrin	+	+	-	+	-	^70^
36	9.087	593.1489	285*, 255, 151	595.167	287*	C_27_H_30_O_15_	3.89	Kaempferol-3-*O* rutinoside	-	+	+	+	-	^68^
37	9.11	463.0856	301*,** 300**	465.1022	303*	C_21_H_20_O_12_	5.6	Quercetin-3-*O*-hexoside	+	-	-	+	-	^60^
38	9.375	447.0918	447, 401, **285**, 284*, 255, 197, 151	449.1075	287*	C_21_H_20_O_11_	3.31	Kaempferol-*O*-hexoside	+	+	+	+	+	^60^
39	9.418	447.0918	301, 300*			C_21_H_20_O_11_	4.65	Quercetin-*O*-deoxyhexose	-	+	-	-	-	^27^
40	9.471	417.2118	417, 284*, 255, 209			C_20_H_34_O_9_	2.88	Kaempferol-*O*- pentoside	+	+	+	+	+	^32^
41	9.494	303.0489	285, 125*			C_15_H_12_O_7_	6.99	Taxifolin	+	-	-	-	-	^71^
42	9.584	431.0952	268*,** 269**	433.1120	271*	C_21_H_20_O_10_	7.34	Apigenin-*O*-hexoside	+	-	-	-	-	^47^
43	9.808	463.1705	463, 301*,** 300**, 194, 150, 106	465.1022	303*	C_21_H_20_O_12_	5.6	Quercetin-*O*-hexoside	-	+	+	+	+	^27^
44	9.937	431.0964	285*			C_21_H_20_O_10_	4.56	Kaempferol-3-*O*-*α*-L-rhamnoside	-	+	-	-	-	^27^
45	10.571	461.1086	299*,** 298**	463.122	301*	C_22_H_22_O_11_	0.73	Chrysoeriol-7-*O*-hexoside	+	-	-	-	-	^72^
46	10.713	593.1231	447, 284*,** 285**, 163, 119	595.1435	287*	C_30_H_26_O_13_	6.84	Kaempferol-*p*-coumaroyl hexoside	+	-	-	-	-	^32^
47	10.919	285.0403	239, 217,199, 169, 151, 133, 110, 80	287.0549	153	C_15_H_10_O_6_	0.56	Kaempferol	+	-	+	-	+	^32^
48	11.392	301.0609	301	303.1944	161	C_16_H_14_O_6_	7.16	Hesperetin	+	-	-	-	-	^73^
49	11.602	269.0443	117*	271.059	153, 119*	C_15_H_10_O_5_	4.62	Apigenin	+	+	+	-	-	^47^
50	12.152	329.063	**314**, 299,271*	331.0810	316, 301, 273	C_17_H_14_O_7_	11.14	Cirsiliol	+	-	-	-	-	^74^
51	12.64	739.1618	**593**, 285, 284*	741.1796	287	C_39_H_32_O_15_	6.81	Di-*O*-*p*-coumaroyltrifloin	+	-	-	-	-	^75^
52	12.7	477.1034	315	479.1179	317	C_22_H_22_O_12_	0.94	Isorhamnetin hexoside	+	-	-	-	-	^76^
Biflavonoids														
53	11.728	537.0812	375*	539.0812	**377**	C_30_H_18_O_10_	2.45	Cupressuflavone	-	+	+	+	+	^38^
54	11.68	537.0821	443, 375*, 331			C_30_H_18_O_10_	1.15	Biapigenin	+	-	-	-	-	^38^
55	11.993	537.0814	375*	539.0814	**377**	C_30_H_18_O_10_	2.45	Amentoflavone	-	+	+	+	+	^38^
56	12.256	537.0814	519, 443, 417, 375*, 331	539.0814	**377**	C_30_H_18_O_10_	2.45	Robustaflavone	-	+	+	+	+	^38^
57	13.578	537.0814	537, 375*	539.0814	377*	C_30_H_18_O_10_	2.45	Hinokiflavone	-	+	+	+	+	^38^
58	14.658	551.0964	536, 375*, 283, 255			C_31_H_20_O_10_	3.57	Monomethoxylbiflavone	-	+	+	+	+	^38^
59	14.861	551.0964	507, 375*, 331, 217			C_31_H_20_O_10_	3.57	Monomethoxylbiflavone	-	+	-	+	+	^38^
Lignans														
60	8.552	521.1995	**359**, 83*			C_26_H_34_O_11_	6.39	Lariciresinol hexoside	+	-	-	-	-	^75^
61	9.559	519.1855	357*, 342			C_26_H_32_O_11_	3.24	Unknown lignan hexoside	-	-	+	+	+	-
62	9.721	361.1653	179, 165, 125			C_20_H_26_O_6_	1	Secoisolariciresinol	+	-	+	-	-	^77^
63	11.554	357.1325	**342**, 221, 137, 83*	359.1486	137	C_20_H_22_O_6_	5.2	Matairesinol	+	+	+	+	+	^38^
Diterpene acids														
64	12.397	349.2016	331, 261*			C_20_H_30_O_5_	1.28	Dehydroxydehydro abietic acid derivative	+	-	-	+	+	^64^
65	14.114	329.1735	285			C_20_H_26_O_4_	7.07	Carnosol	+	-	-	-	-	^75^
66	14.883	333.2066	301			C_20_H_30_O_4_	0.7	Abietic acid derivative	+	-	-	+	-	^64^
67	15.688	321.2424	303, 277*, 259	323.26	**305**, 277, 69	C_20_H_34_O_3_	3.47	Imbricatolic Acid	-	+	+	+	-	^41^
68	15.789	331.1918	313			C_20_H_28_O_4_	1.15	dihydroxydehydro abietic acid	+	+	+	+	+	^64^
69	16.40			301	286	C_20_H_28_O_2_	−3.64	Abietatrienoic acid	+	-	-	-	-	-
70	18.11	301.2159	**257**			C_20_H_30_O_2_	4.64	Isopimaric acid	+	-	-	+	+	^75^
71	18.113	301.2161	**255**, 239, 220, 205*			C_20_H_30_O_2_	4.64	Communic acid	+	+	+	+	+	^78^
Fatty acids and their amides														
72	11.182	327.2163	309, 291, 229, 221, 211,171*			C_18_H_32_O_5_	4.26	Trihydroxy-octadecadienoic acid	+	+	+	+	+	^25^
73	11.594	329.2322	299, 229, 211*			C_18_H_34_O_5_	3.48	Trihydroxy-octadecaenoic acid	+	+	+	+	+	
74	11.682	329.2328	299, 271, 229, 211*, 171, 155			C_18_H_34_O_5_	1.66	Pinellic acid	+	-	-	-	-	^79^
75	12.053	287.2212	269*, 171			C_16_H_32_O_4_	5.49	Dihydroxy-palmitic acid	-	-	+	+	+	^60^
76	14.302			274.2753	256*, 230	C_16_H_35_NO_2_	−5.28	2-Amino-1,3-hexadecanediol	+	+	+	+	+	^80^
77	15.343	293.2107	275, 96*	295.226	277, 98	C_18_H_30_O_3_	5.16	Hydroxyoctadecatrienoic acid	+	+	+	+	+	^25^
78	15.799	483.2713	439, 255*			C_29_H_40_O_6_	8.49	Palmitic acid derivative	+	+	+	+	+	^60^
79	16.01	271.2275	253, 225*			C_16_H_32_O_3_	5.03	Hydroxy-palmitic acid	+	+	+	+	+	^60^
80	16.144	295.2265	277*,171			C_18_H_32_O_3_	4.96	Hydroxy-octadecadienoic acid	+	+	+	+	+	^25^
81	18.60	271.23	225*			C_16_H_32_O_3_	6.5	Hydroxy hexadecenoic acid	+	+	+	+	+	-
82	18.64	301.2159	286			C_20_H_30_O_2_	4.64	Eicosapentaenoic acid	+	+	+	+	+	^81^
83	18.904	277.2158	233, 215, 129*			C_18_H_30_O_2_	4.33	Linolenic acid	+	+	+	-	+	^81^
84	19.051	355.3204	337, 309*			C_22_H_44_O_3_	3.84	Hydroxy-docosanoic acid	-	-	-	+	+	^60^
85	19.044	383.1867	337,84*			C_24_H_48_O_3_	3.3	Hydroxy-tetracosanoic acid	-	+	-	+	+	^60^
86	19.103			228	102, 88*	C_14_H_29_NO	3.33	Myristamide	+	+	+	+	+	
87	19.163			254	121	C_16_H_31_NO	−5.76	Hexadecenamide	+	+	+	+	+	^81^
88	19.504			280	121, 109, 95	C_18_H_33_NO	−5.4	linoleamide	+	+	+	+	+	^81^
89	20.323			268	250, 226	C_17_H_33_NO	−5.27	Unknown fatty acid amide	+	+	+	+	+	-
90	21.122			310	293, 275	C_20_H_39_NO	2.29	Eicosenoic acid Amide	+	+	-	+	+	^62^
91	21.238			338	321, 303	C_22_H_43_NO	2.99	Docosenoic acid Amide (docosenamide)	+	+	+	+	+	^62^
92	21.707			282	265, 149, 135, 121	C_18_H_36_NO	−0.32	Oleamide	+	+	+	+	+	^81^
Others														
93	2.599	165.0552	150, 96			C_9_H_10_O_3_	3.12	3-Hydroxyphenylpropionic acid	-	+		-	-	^67^
94	2.882	181.0498	165, 149, 105			C_9_H_10_O_4_	4.57	3-Hydroxy-3-(3-hydroxyphenyl) propionic acid	+	-	-	-	-	^67^
95	3.89	359.1333	181, 59			C_16_H_24_O_9_	4.04	Junipediol A 8-glucoside (phenolic glycosides)	-	+	-	+	-	^82^
96	6.71	461.1288	167			C_19_H_26_O_13_	2.74	Saccharumoside C/D (tannin)	-	-	-	+	-	^82^
97	7.354	167.0345	152, 108			C_8_H_8_O_4_	2.87	3,4-Dihydroxyphenylacetic acid (Hydroxyphenylacetic acids class)	+	+	-	+	-	^67^
98	9.598	327.1437	165			C_16_H_24_O_7_	4.04	Betuloside (phenylpropanoid) natural phenol	-	-	-	+	+	^70^
99	11.34	181.0498	166, 151, 138			C_9_H_10_O_4_	4.57	Syringaldehyde (organic aldehyde)	-	-	-	+	+	^27^

+: Present, -: Absent, *: Base peak (“The most intense peak”), Bold numbers: significant fragments, PC: *Pinus canariensis* C.Sm. extract, CL: *Cupressus lusitanica* Mill. extract, Fra. MC. CL: methylene chloride fraction of CL extract, CA: *Cupressus arizonica* Greene extract, Fra. MC. CA: methylene chloride fraction of CA extract.

#### Acids and their derivatives

##### a. Organic acids

Organic acids are organic compounds with simple structures, containing one or more carboxylic groups. In plant biology, these acids act as pH modifiers, chelators for binding metals, and as carbon sources for microbes^21^.

In the three extracts of PC, CL, CA and their most active fractions (Fra. MC. CL and Fra. MC. CA), peaks **2** and **4** (Table 3, Supplementary Fig. S2 online) were annotated in the negative ESI mode as quinic and shikimic acids. They were detected at [M–H]^–^ *m/z* 191.0552 and 173.0434 with the molecular formula C_7_H_12_O_6_, and C_7_H_10_O_5_, respectively. They showed characteristic fragment ions after the loss of water [M–H–H_2_O]^–^ at *m/z* 173 and 155, respectively, and they were previously reported in the *Cupressus* genus^9^. Recent studies have shown that various organic acids exhibited antivirulence mechanisms at sublethal concentrations by interfering with quorum-sensing systems in bacteria^22^.

##### b. Phenolic acids

Phenolic acids are secondary plant metabolites with one or more hydroxyl groups, and a carboxylic group attached to the aromatic ring. They possess a variety of biological activities, such as antimicrobial, antioxidant, and antiviral properties^23,24^. In the negative and positive ESI modes (Table 3), protocatechuic acid **(7)** [*m/z* 153.0182 and 155.0336, respectively (C_7_H_6_O_4_)] showed an intense ion at *m/z* 109 and *m/z* 111 after the loss of CO_2_ [M–H–44]^–^, [M + H–44]^+^, respectively (Table 3, Supplementary Fig. S3 online). Similarly, in the negative ESI mode, protocatechuic acid-hexoside **(12)** [*m/z* 315.0707 (C_13_H_16_O_9_)] showed the same fragment ion at *m/z* 109 after the successive losses of the attached hexose moiety and CO_2_ [M–H–162–44]^–^(Table 3, Supplementary Fig. S3 online).

Furthermore, gallic acid (**3**) (Table 3) [*m/z* 169.0137, (C_7_H_5_O_5_)] and methyl gallate **(13)** (Table 3) [*m/z* 183.0282, (C_8_H_8_O_5_)] were annotated in the three extracts and their fractions. They showed fragment ions [M–H–44]^–^ at *m/z* 125 and [M–H–15]^–^ at *m/z* 168 indicate loss of CO_2_ and methyl group, respectively.

In accordance with the reported data^25^, dihydroxy benzoic acid methyl ether-*O*-hexoside (10) (Table 3) [*m/z* 329.0869 (C_14_H_18_O_9_)] and 5-methoxy salicylic acid **(11)** (Table 3) [*m/z* 167.0341 (C_8_H_8_O_4_)] were annotated in the CL extract in negative ESI mode. After fragmentation, peak **10** showed a loss of hexose [M–H–162]^–^ at *m/z* 167 and then a loss of a methyl group [M–H–162–15]^–^ at *m/z* 152, while peak **11** showed a loss of a methyl group [M–H–15]^−^ at *m/z* 152.

Moreover, caffeoyl-*O*-hexoside (14) (Table 3) [*m/z* 341.0871 (C_15_H_18_O_9_)] and caffeic acid (18) (Table 1) [*m/z* 179 (C_9_H_8_O_4_)] were annotated in the PC extract. On the other hand, peak 16 (Table 1) [*m/z* 337.0915 (C_16_H_18_O_8_)] was detected in CA and CL extracts. It presented two characteristic fragment ions in negative ESI mode, at *m/z* 163 and 191, due to the loss of coumaroyl and quinic acid moieties, respectively.

Hydroxycinnamic acid derivatives were previously reported to exhibit antimicrobial activity against different strains of Gram-positive and Gram-negative bacteria^26^. In all samples, in the negative and positive ESI modes, *p*-coumaric acid (**17**) (Table 3, Supplementary Fig. S4 online) [*m/z* 163.0393 and 165.0544, respectively (C_9_H_8_O_3_)] was detected. It was clearly characterized by its fragment ion at *m/z* 119 and *m/z* 121 corresponding to loss of CO_2_ [M–H–44]^−^ and [M + H–44]^+^, respectively. These findings were consistent with previously reported literature related to *Cupressus* species^9^. Additionally, compared to previous LC-MS reported data, 3,5-di-*p*-coumaroyl quinic acid (**5**) (Table 3) [*m/z* 482.745 (C_25_H_22_O_10_)] was identified. It revealed, in the negative ESI mode, characteristic fragment ions at *m/z* 337, 319, and 163^27^.

#### Catechins and their derivatives

Procyanidins are oligomers or polymers of monomeric flavan-3-ols (catechin and epicatechin molecules) that are a byproduct of the flavonoid biosynthetic pathway. They are phytochemical classes previously detected in different coniferous plants^28^ and possess different biological activities such as antioxidant, anti-inflammatory, and antimicrobial effects^29^.

Among the annotated catechins, in negative ESI mode, were epi/gallocatechin (**22**) (Table 3) [*m/z* 305.07 (C_15_H_14_O_7_)] and epi/catechin (**27**), in the negative and positive ESI modes, (Table 3) [*m/z* 289.0707, 291.0874, respectively (C_15_H_14_O_6_)]. They showed characteristic fragment ions at *m/z* 125 and 245 corresponding to loss of C_9_H_8_O_4_, and CO_2_, respectively, and were detected herein in PC, CL, and CA extracts. Peak **24** (Table 3, Supplementary Fig. S5 online) [*m/z* 451.1221 (C_21_H_24_O_11_)] was also identified in negative ESI mode in these extracts as catechin-7-*O-β*-D-glucopyranoside, based on the presence of fragment ion at *m/z* 289 after the loss of the linked hexose unit. Peak **25** (Table 3, Supplementary Fig. S5 online), in negative and positive ESI modes, [*m/z* 577.1334 and 579.1527, respectively (C_30_H_26_O_12_)] and was annotated as procyanidin B, showing a fragment ion at *m/z* 289 [M–H–288]^−^ and *m/z* 291 [M + H–288]^+^, respectively, due to the cleavage of the inter-flavan linkage. This metabolite was previously reported in the *Cupressus* genus^9^. The results clearly illustrated that the abundance of catechins and their derivatives in the two *Cupressus* species was higher than in the *Pinus* one (Fig. 3; Table 3).

#### Flavonoids and their glycosides

Flavonoids are polyphenolic plant secondary metabolites. They are synthesized by the polypropanoid pathway, which starts with phenylalanine. In plants, they play a role in protection from microbes and insects, serve as antifungal plant agents, and fight against mammalian herbivory^24^. Flavonoids may be found as aglycones or in glycosidic form (O or C-linked bound to one or more sugar moieties)^30^.

##### a. Flavonols

Eleven flavonols and flavonol glycosides were annotated, mainly quercetin and kaempferol glycosides. Peaks 30, 32, 36, 37, 38, 42, 43, and 46 in both negative and positive ESI modes and peaks 39, 40, and 44 (Table 3) in negative ESI mode only. They were recognized based on the loss of rhamnosyl (−146) pentosyl (−132), or hexosyl (−162) moieties to generate their characteristic aglycones of quercetin (301) and kaempferol (285)^31^.

In the CA and CL extracts, rutin (32) (Supplementary Fig. S6 online) showed [M–H]^–^ and [M + H]^+^ ions [*m/z* 609.1437 and 611.1615, respectively (C_27_H_30_O_16_)] and kaempferol-3-*O-*rutinoside (36) (Supplementary Fig. S6 online) [*m/z* 593.1489 and 595.167, respectively (C_27_H_30_O_15_)]. They revealed fragment ions in the negative ESI mode [M–H–146–162]^−^ at *m/z* 301 and [M–H–308]^−^ at *m/z* 285, respectively, and in the positive ESI mode mode [M + H–146–162]^+^ at *m/z* 303 and [M + H–308]^+^ at *m/z* 287, respectively. These fragments corresponded to loss of sugar units^32^.

Similarly, in the CL extract, kaempferol rhamnosyl-hexoside (30) [*m/z* 593.1489 and 595.1678 in both ESI modes (C_27_H_30_O_15_)], quercetin-*O*-deoxyhexose (39) [*m/z* 447.0918 in negative ESI mode (C_21_H_20_O_11_)] and kaempferol-3-*O*-*α*-L-rhamnoside (44) [*m/z* 431.0964 in negative ESI mode (C_21_H_20_O_10_)] (Table 3) were annotated. They revealed intense ions at 285 *m/z* [M–H–146–162]^−^, 301 *m/z* [M–H–146]^−^, and 285 *m/z* [M–H–146]^−^, respectively, indicating that the aglycone was quercetin or kaempferol.

Additionally, peaks 37, 38, 40, and 43 (Table 3) were detected, namely as quercetin-3-*O*-hexoside (37) [*m/z* 463.0856 and 465.1022 in both ESI modes (C_21_H_20_O_12_)], kaempferol-*O*-hexoside (38) [*m/z* 447.0918 and 449.1075 in both ESI modes (C_21_H_20_O_11_)], kaempferol-*O*-pentoside (40) [*m/z* 417.2118 in positive ESI mode (C_20_H_34_O_9_)], and quercetin-*O*-hexoside (43) [*m/z* 463.1705 and 465.1022 in both ESI modes (C_21_H_20_O_12_)], in accordance with previous reported data^9,31^.

Among other annotated flavonol glycosides, showing conjugation with coumaroyl moieties was di-*O*-*p*-coumaroyl trifolin (51) [*m/z* 739.1618 and 741.1796 in both ESI modes (C_39_H_32_O_15_)] that was exclusively detected in the PC extract. Its MS/MS spectrum displayed a characteristic fragment ions at *m/z* 593 in negative ESI mode due to the loss of a *p*-coumaroyl unit, as well as at *m/z* 285 and *m/z* 287 in the negative and positive ESI modes, respectively, corresponding to the kaempferol aglycone.

##### b. Flavanones

Two flavanones were detected in the negative ESI mode for the first time in the CL extract, eriodictyol-7-*O*-glucoside (31) (Table 3) [*m/z* 449.1078 (C_21_H_22_O_11_)] and naringenin-7-*O*-hexoside (34) (Table 3) [*m/z* 433.1699 (C_21_H_22_O_10_)]. Their fragmentation behavior showed characteristic ions [M–H–162]^−^ at *m/z* 287 and 271, respectively which indicated a loss of a 162 (hexose) moiety.

##### c. Flavones

Apigenin, peak 49 (Table 3) [*m/z* 269.0443 and 271.0599 in both ESI modes (C_15_H_10_O_5_)], was detected in the PC and CL extracts, in agreement with previously reported data^33^.

##### d. Flavan-3-ol

Peaks 33 (Table 3) [*m/z* 465.101 (C_21_H_22_O_12_)] and 41 (Table 3) [*m/z* 303.0489 (C_15_H_12_O_7_)] were exclusively detected in the negative ESI mode in the PC extract. The fragment ion, [M–H–162]^–^ at *m/z* 303, was formed after the loss of a hexose sugar in peak 33. Comparing both mass spectra and considering previous reports^34^, they were annotated as taxifolin-O-hexoside and taxifolin, respectively.

#### Biflavonoids

Biflavonoids are dimers of flavone-flavone, flavone-flavonone, and flavone-flavonone subunits, as well as dimers of chalcones and isoflavones in rare cases^35^. They are considered one of the major reported chemical classes in conifers, in particular, the *Cupressus* species^36^. Biflavonoids exhibit significant biological activities, such as antimicrobial, anti-inflammatory, antioxidant, and cytotoxic properties. As depicted in Table 3, five biflavonoids, peaks 53–57, were detected in both the negative and positive ESI modes. In addition, two methoxybiflavones isomers were annotated in negative ESI mode, peaks 58 and 59.

Four out of the five detected biflavonoids were found in high abundance in CL and CA extracts in both ESI modes (53-55-57), while in PC extract was only detected one biflavonoid in negative ESI mode (4) (Table 3; Fig. 3). These bioflavonoids were, cupressuflavone (53) (Supplementary Fig. S7 online) [M–H]^–^ and [M + H]^+^ ions at [*m/z* 537.0812 and 539.0812, (C_30_H_18_O_10_)], biapigenin (54) [M–H]^–^ ion at [*m/z* 537.0812 (C_30_H_18_O_10_)], amentoflavone (55) [*m/z* 537.0814 and 539.0812, in both ESI modes (C_30_H_18_O_10_)], robustaflavone (56) [*m/z* 537.0814 and 539.0812, in both ESI modes (C_30_H_18_O_10_)], and hinokiflavone (57) [*m/z* 537.0814 and 539.0812, in both ESI modes (C_30_H_18_O_10_)]. They all showed a characteristic fragment ion after the breakdown of the linkage of the dimer units [M–H–162]^−^ at *m/z* 375 and [M + H–162]^+^ at *m/z* 377 in the negative and positive ESI modes, respectively. These assignments agreed with the reported literature^37^. In addition, in the negative ESI mode, two methoxybiflavones isomers (58,59) (Table 3) [*m/z* 551.0964, (C_31_H_20_O_10_)] showed fragment ions at *m/z* 375, resulting from the breakdown of the linkage of the dimer units [M–H–162]^−^. All the detected biflavonoids were previously reported in the *Cupressus* genus but, to the best of our knowledge, they were identified for the first time here in CL extract^37^.

##### Lignans

Lignans are a type of secondary metabolite that belongs to the phenylpropanoids (phenylpropane derivatives) class, characterized by a tricarbon chain connected to the aromatic nucleus. They are generated by dimerization of two phenylpropanoid units with varying degrees of oxidation and aromatic moiety substitution patterns. Lignans possess a wide range of biological activities, such as antioxidant, antibacterial, antiviral, anti-fungal, and insecticidal properties^38^. Four lignans were annotated in the negative mode, particularly in PC and CA extracts. Lariciresinol hexoside **(60)** (Table 3, Supplementary Fig. S8 online) [*m/z* 521.1995, C_26_H_34_O_11_], was exclusively detected in the PC extract. It exhibited a characteristic fragment ion at *m/z* 359, indicative of the loss of a hexosyl moiety, [M–H–162]^–^. Matairesinol (**63**) (Table 3) was the only lignan detected in the three extracts in both ESI modes ([M–H]^–^ and [M + H]^+^ ions at *m/z* 357.1325 and 359.1486 (C_20_H_22_O_6_), respectively). Its spectrum showed a fragment ion at *m/z* 342 resulting from the loss of a methyl group [M–H–15]^−^, as previously reported in the *Cupressus* genus^37^.

#### Diterpene acids

Diterpenoids are isoprenoids with twenty carbon skeletons. Some diterpenoids play a resistance role against pests or pathogens. For example, diterpene resin acids are considered as major components of the oleoresin defense system in conifer trees, such as in the Pinaceae family^39^. Eight diterpene acids were annotated in the extracts and their fractions (64–71) (Table 3). For example, peak 67 (Supplementary Fig. S9 online) (*m/z* 321.2424 and 323.2600 in both ESI modes (C_20_H_34_O_3_)) was identified as imbricatolic acid and was previously reported in the *Cupressus* genus^40^. In the negative ESI mode, mass spectrum presented a characteristic fragment ion at *m/z* 277 due to the loss of water and further loss of CO [M–H–18–28]^−^.

Other two diterpene acids, were detected in the negative ESI mode, peaks 70 and 71 (Table 1). In detail, peak 70 [*m/z* 301.2159, (C_20_H_30_O_2_)] was identified as isopimaric acid, based on its fragment ion at *m/z* 257, due to the loss of a CO_2_ from the carboxylic acid group [M–H–44]^−^. Peak 71 [*m/z* 301.2161, (C_20_H_30_O_2_)] was annotated as communic acid, presenting a fragment ion at *m/z* 255 due to the loss of oxygen and 2 methyl groups [M–H–16–30]^−^. Communic acid was previously reported in *Cupressus* genus^40^.

#### Fatty acids and their amides

Fatty acids are carboxylic acids with an aliphatic chain that can be either saturated or unsaturated (monounsaturated or polyunsaturated). Different types of fatty acids, such as oleic and lauric acids, were reported as lipase inhibitors^41^. Twenty-one fatty acids, fatty acid amides, and hydroxy fatty acids were annotated in both ESI modes in the extracts and their fractions)72–92) (Table 3), eluting in the last part of the chromatograms (Fig. 3) (Rt, 11.2–21.7 min). Distinctly fragment ions, resulting from the removal of water or methyl groups were observed in their fragmentation patterns. For instance, peak 72 (Table 3, Supplementary Fig. S10 online) [*m/z* 327.2163, (C_18_H_32_O_5_)] and 73 (Table 1) [*m/z* 329.2322, (C_18_H_34_O_5_)], were annotated in the negative ESI mode, and showed the successive losses of water moieties (−18). A mass difference of 2 m/z units between both compounds was indicative of an extra double bond and they were annotated as trihydroxy-octadecadienoic (72) and trihydroxy-octadecaenoic (73) acids. It is worth noting that fatty acid amide peaks (86–92) (Table 3) were exclusively detected in the positive ESI mode. Their identification was based on the even m/z values of their pseudomolecular ions and the loss of ammonia molecules (−17).

### Identification of the isolated compounds using NMR and TLC

Four compounds were isolated from the methanolic extracts of CL and CA aerial parts for the first time, namely, epicatechin (compound C1, peak 23 in Table 3), rutin (compound C2, peak 32 in Table 3), 3,5-di-*p*-coumaroylquinic acid (compound C3, peak 5 in Table 3), and cupressuflavone (compound C4, peak 53 in Table 3). The chemical structures of the isolated compounds are presented in (Supplementary Fig. S11 online). These identifications were confirmed by NMR and TLC analyses, using standards when they were commercially available.

Compound C1; (epicatechin)^42^; white needles^1^;H NMR (400 MHz, DMSO-d_6_); δ 2.39 (1 H, *dd*, *J* = 16, and 8 Hz, H_β_−4), 2.65 (1 H, *dd*, *J* = 16, and 4 Hz, H_α_−4), 3.83 (1 H, m, H-3), 4.47 (1 H, *d*, *J* = 8 Hz, H-2), 5.69 (1 H, *d*, *J* = 4 Hz, H-6), 5.81 (1 H, *d*, *J* = 4 Hz, H-8), 6.58 (1 H, *d*, *J* = 8.2 Hz, H-5`), 6.68 (1 H, *dd*, *J* = 8.2 and 4 Hz, H-6`), 6.72 (1 H, *d*, *J* = 4 Hz, H-2`). The identity was confirmed by co-chromatography with epicatechin analytical standard on TLC, having the same color and R_f_ value 0.56 in MC: Me (8:2, v: v).

Compound C2; (rutin)^43^; yellow amorphous powder^1^;H NMR (400 MHz, DMSO-d6); δ 1.61 (3 H, *d*, *J* = 8, H-6```), 3.38–3.56 (*m*, sugar protons), 4.39 (1 H, *d*, *J* = 4, H-1```), 5.34 (1 H, *d*, *J* = 4, H-1``), 6.18 (1 H, *d*, *J* = 2, H-6), 6.37 (1 H, *d*, *J* = 2, H-8), 6.85 (1 H, *d*, *J* = 12, H-5`), 7.53 (1 H, *d*, *J* = 4, H-2`), 7.56 (1 H, *dd*, *J* = 12,4, H-6`).

^13^C-NMR (400 MHz, DMSO-d6); *δ* 18.18 (C-6```), 67.80-76.69 (carbon sugars), 94.44 (C-8), 101.08 (C-1``), 101.71 (C-1```), 115.68 (C-2`), 116.60 (C-6 `), 133.82 (C-3), 145.25 (C-3`), 149.05 (C-4`), 156.81 (C-5), 161.39 (C-7), 177.71 (C-4). The identity was confirmed by co-chromatography with rutin analytical standard on TLC, having the same color and R_f_ value 0.3 in MC: Me (8:2,v/v).

Compound C3; (3,5-di-*p*-coumaroylquinic acid)^44^; white amorphous powder^1^, H NMR (400 MHz, DMSO-d6); *δ* 2.53 (2H, *m*, H-6), 2.56 (2H,*brs*, H-2), 3.72 (1H, *brs*, H-5), 4.19 (1H, *brs*, H-4), 5.34–5.35 (2H, *m*, H-3, H-5), 6.32,6.36 (1H x 2, *d* x 2, H-8` x 2), 6.78,6.81 (1H x 2, *br d*, H-5` x 2), 7.55,7.57 (1H x 2, *brs*, H-2` x 2),7.57, 7.58 (1H x 2, *d*, *d* x2, H-6` x 2, *J* = 8), 7.52, 7.56 (1H x 2, *d* x2, H-7`x 2, *J*_7’,8’_ = 16).

^13^C-NMR (400 MHz, DMSO-d6); δ 36.3, 36.9 (C2, C-6), 69.7 (d, C-4), 70.6, 70.8 (d x 2, C-3. C-5), 73.56 (s, C-1), 116.2 (x4, C3`, C5`), 125.6, 125.7(C1`, x2), 130.6, 130.7 (C-2`x2), 144.6, 144.7 (C-7` x 2), 160.1, 160.2 (C-4` x 2), 166.4, 166.8 (C-9` x 2), 179.97 (C-7).

Compound C4; (cupressuflavone)^45^; yellow amorphous powder^1^;H NMR (400 MHz, DMSO-d6); δ 6.42 (1 H, *s*, H-6, H-6``), 6.54 (1 H, *s*, H-3, H-3``), 6.71 (2 H, *d*, *J* = 8, H-3`, H-5`, H-3```, H-5```) 7.35 (4 H, *d*, *J* = 8, H-2`, H-6`, H-2```, H-6```).

^13^C-NMR (400 MHz, DMSO-d6); δ 98.8 (C-6, C-6``), 98.6 (C-8, C-8``), 102.55 (C-3, C-3``), 102.90 (C-10, C-10``), 115.68 (C-3`, C-5`, C-3```, C-5```), 121.48 (C-1`, C-1```), 127.81 (C-2`, C-6`, C-2```, C-6```), 154.46 (C-9, C-9``), 161.09 (C-4`, C-4```), 161.3 (C-5, C-5``), 162.52 (C-7, C-7``), 167.6 3 (C-2, C-2``), 182.01 (C-4, C-4``).

Noteworthy, epicatechin (C1), a flavanol from the flavan-3-ol family commonly found in green tea, was previously identified in the methanol extract of *C. macrocarpa* root^46^. It was also reported that several components, such as catechin and epicatechin, of the aquous extract of *P. pinaster* bark could contribute to its antibacterial activity against clinical isolates of *A. baumannii*^47^.

Cupressuflavone (C4) is a unique biflavonoid compound composed of two apigenin molecules linked by a C-C bond. It was previously identified in the methanolic extract of *C. macrocarpa* leaves and was reported as a main chemotaxonomic marker for *Cupressus* species^48^. Additionally, it was previously isolated, along with rutin (C2), from the leaves of different *Cupressus* species (*C. sempervirens*)^49^. Rutin has been shown to inhibit the nucleic acid synthesis of *E. coli.*^50^.

Importantly, flavonoids and biflavonoids were reported to exhibit antibacterial and antivirulence activities. In addition, the biofilm formation of *S. carnosus* was strongly suppressed by tetrahydroamentoflavone^51^.

### Lipase assay for the isolated compounds

**Fig. 4:**
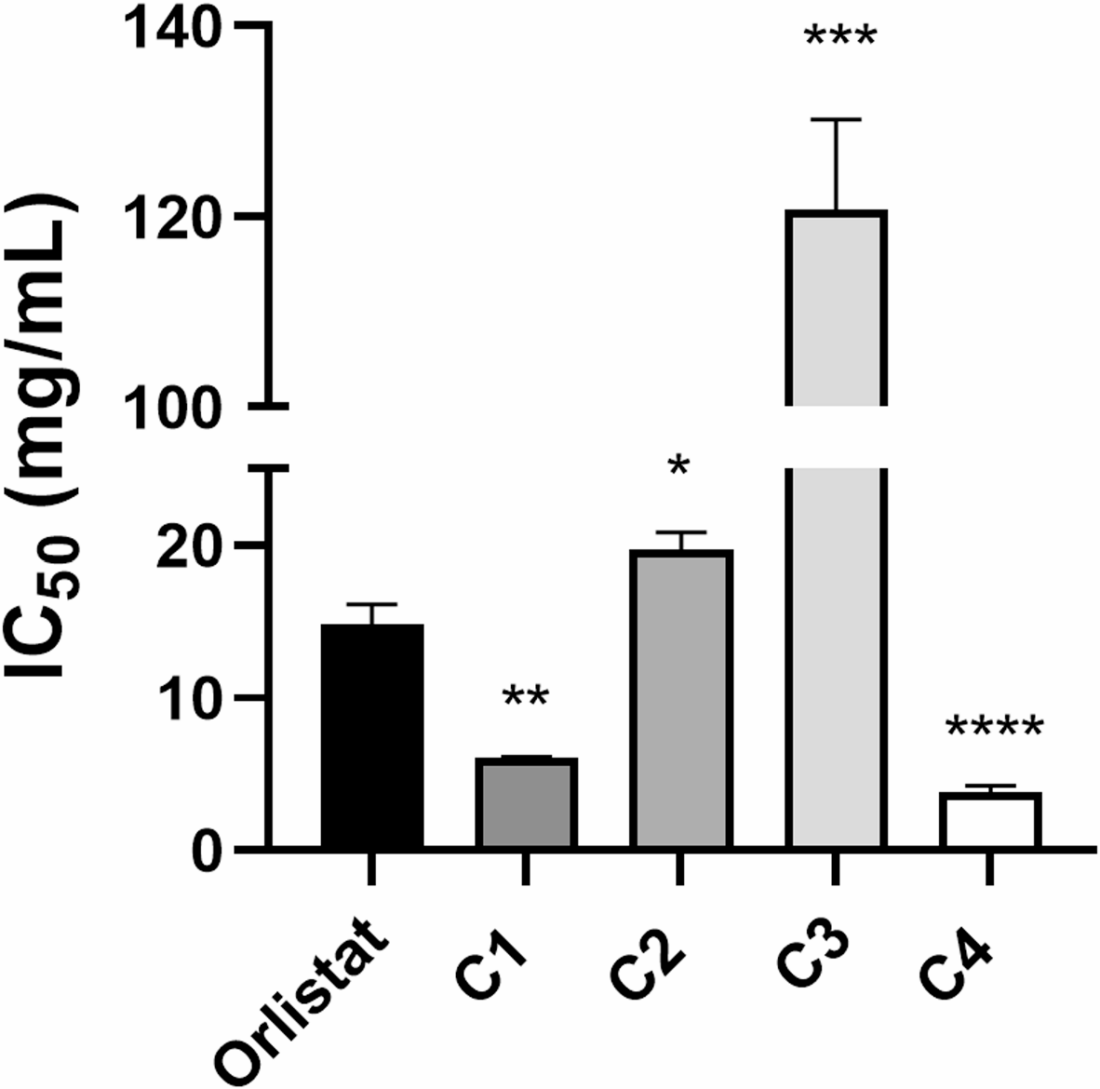
IC_50_ of the four isolated compounds (C1, C2, C3, and C4) against *A. baumannii* lipolytic activity. Values are presented as mean ± SEM of three replicates, and error bars are the SEM. Values were significantly different as compared to the positive control, orlistat, by the student’s *t*-test, *p < 0.05, **p < 0.01, ***p < 0.001, and ****p < 0.0001.

The IC_50_ values for the four isolated compounds were determined by extrapolation from the graph generated using the tested concentration (Fig. 4; Table 4). Cupressuflavone (C4), showed the lowest IC_50_ value (3812 ± 450 µg/mL) among the isolated compounds (Table 4), which was also significantly lower than that of the positive control, orlistat (14835 ± 920 µg/mL) (Fig. 4). Indeed, cupressuflavone was approximately 3.89 times more potent against *A. baumannii* lipases compared to orlistat. By comparing with previously tested compounds, a previous in-silico study demonstrated the anti-lipolytic potential of glucovanillin by investigating its interaction with a purified lipase from *A. radioresistens* PR8^41^. Moreover, Farnesol, an acyclic sesquiterpene alcohol mostly found in the essential oils of different plants, is another naturally occurring anti-*Staphylococcus aureus* agent that may lessen its pathogenicity by inhibiting its lipases with an IC_50_ value of 0.57 mM^52^. In addition, rhodomyrtone, a pure substance isolated from *Rhodomyrtus tomentosa* leaves, dramatically reduces *Propionibacterium acne*s’ ability to produce lipases, a key factor in the progression of acne vulgaris^53^.

**Table 4:** IC_50_ values against *A. baumannii* lipolytic activity for Orlistat (positive control) and the four isolated compounds from the methanolic extracts of CL and CA.

Compound	IC_50_ (µg/mL) (Mean ± SEM)
Epicatechin (**C1**)	6090 ± 60
Rutin (**C2**)	19,721 ± 1120
3,5-di-*p*-coumaroylquinic acid (**C3**)	120,756 ± 9420
Cupressuflavone (**C4**)	3812 ± 450
Orlistat (control)	14,835 ± 920

IC_50_: half-maximal inhibitory concentration. The studied concentration ranges were 16–2048 µg/mL for C3, C4, and orlistat and 16–512 µg/mL for C1 and C2.

### Multivariate data analysis. Correlation of metabolites with the lipolytic activity

**Fig. 5:**
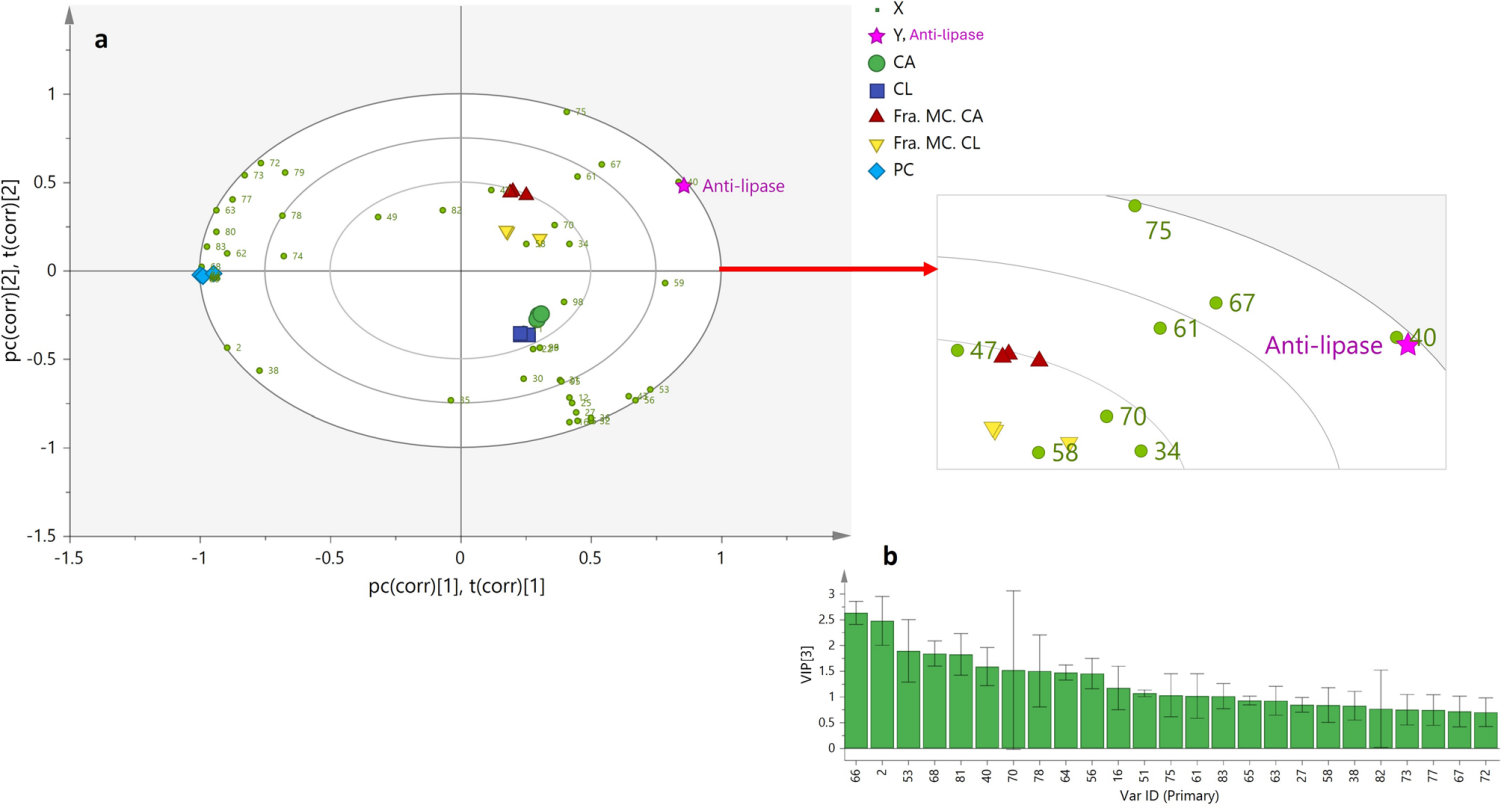
(**A**) PLS biplot describing the correlation between the identified metabolites in PC, CA, and CL extracts and their most active fractions (Fra. MC. CL, Fra. MC. CA) and lipolytic activity inhibition. See Table 3 for the metabolite assignments. (**B**) Twenty-five metabolites with variable importance in the projection (VIP) values ≥ 0.7.

PLS is a supervised multivariate “regression technique” that is used to describe the relationship between dependent variables (Y variables) and independent variables or predictors (X variables), with explanatory or predictive purposes. The method is widely used to investigate quantitative structure-activity relationships to investigate the chemical characteristics of the samples (X variables) with their bioactivities (Y variables)^54^. Although multivariate tools like PLS do not directly isolate compounds, they enhance bioassay-guided isolation by identifying metabolites statistically linked to biological activity. This focused strategy streamlines the process by reducing the number of compounds requiring structural elucidation, such as NMR. In this study, multivariate analysis served as a complementary approach to support, rather than replace, conventional isolation methods. In this context, PLS analysis was used to find the correlation between the metabolites identified in PC, CL, CA extracts, along with their most active fractions (Table 3), and lipolytic activity (Table 4). To this end, the intensities of the identified metabolites across samples were considered as the X variables and the lipolytic activity inhibition as the Y variables. Figure 5A shows the PLS biplot for the PLS analysis. The PLS biplot is a combination of the scores and loading plots where the distance of the X and Y variables to the sample clusters indicates the extent of their contribution to the characteristics of the considered cluster. The PLS biplot showed good fitness (R2Y = 0.73) and predictive ability (Q2 = 0.70). The PLS model was validated using regression analysis (Supplementary Fig. S12 online) and 200 permutation tests with Y-intercepts R2 = 0.227 and Q2 = − 0.512, confirming its validity and absence of overfitting (Supplementary Fig. S12 online). As can be observed on the right side of the PLS-biplot, the lipolytic activity inhibition was closely projected to the MC fractions of the CA and CL extracts, suggesting a strong correlation, followed by their respective extracts, CA and CL. In contrast, the PC extract samples were clustered on the far-left side of the plot indicating a weak correlation (Fig. 5A). This agrees with the bioassay results where the MC fractions of CA and CL extracts showed the highest activity, followed by the CA and CL extracts, while the activity of PC extract was statistically significantly lower than for the other samples (Fig. 2B). The VIP approach was used to pinpoint the metabolites that exerted the most significant influence in the PLS model (Fig. 5B). Based on the VIP approach, 25 metabolites contributed the most to discriminate the lipolytic activity inhibition (with a VIP value ≥ 0.7), namely, two phenolic acids (2 & 16), one catechin (27), three flavonoids (38, 40, & 51), three biflavonoids (53, 56, & 58), two lignans (61 & 63), six diterpene acids (64–68, & 70), and eight fatty acids (72–73, 75, 77–78, & 81–83) (Fig. 5B). These metabolites were found to be particularly abundant in the CA and CL extracts and their respective MC fractions.

Notably, the collective presence of these metabolite classes appeared to exert a synergistic effect, contributing to discriminate the anti-lipase activity of the bioactive extracts and their fractions. Some of the discriminant metabolite classes align with previous findings regarding the highly abundant isolated compounds (C1-C4, i.e. 23, 32, 5 & 53), which belong to the catechins, flavonoid glycoside, phenolic acid, and biflavonoid categories, respectively, and have demonstrated significant anti-lipase activity (Table 4). However, only C4 (54) was relevant for discrimination of the studied samples. This approach helped to focus on key features driving the bioactivity, allowing for more targeted downstream isolation efforts. Future studies could integrate this workflow with expanded biological screening and structural characterization to accelerate the discovery of lead compounds.

Overall, targeting the lipases of *A. baumannii* presents a novel approach and underexplored therapeutic strategy, as limited research has been conducted on this specific virulence mechanism. As extensive research focused on the effects of various herbal components on human lipases^11^. However, phenolic acids, such as quinic acid and its derivatives, have demonstrated efficacy in reducing biofilm formation in several foodborne pathogens, including *Staphylococcus aureus*,* Bacillus* spp., *Yersinia enterocolitica*, and *Escherichia coli*^55^. Similarly, catechins, like epicatechin gallate and epigallocatechin, have been reported to disrupt biofilm formation, quorum sensing, and gene expression modulation in *Streptococcus mutans*^56^. Additionally, rutin, a flavonoid glycoside, has demonstrated potent antibacterial and anti-virulence properties by inhibiting bacterial growth and biofilm formation in various antibiotic-resistant bacteria, such as *Pseudomonas aeruginosa* and Methicillin-resistant *Staphylococcus aureus*^57^. Furthermore, biflavonoids have displayed broad-spectrum activity against both Gram-positive and Gram-negative bacteria, with diverse anti-virulence mechanisms, such as suppressing biofilm formation in *Streptococcus pyogenes*^58^. While these findings highlight the potential of natural compounds in targeting bacterial virulence factors, their anti-lipase activity is still unexplored and needs further validation. Prioritizing studies on lipase inhibition mechanisms, synergies with existing antibiotics, and in vivo efficacy will be critical to advancing this therapeutic approach.

## Conclusion

This study is the first to highlight the potential use of methanolic extracts from the aerial parts of three coniferous plants and their fractions in inhibiting the lipolytic activity of *A. baumannii.* This approach could serve as a valuable therapeutic strategy for combating infections caused by this harmful pathogen. The CL and CA extracts demonstrated significantly greater potency than the PC extract, with the Fra. MC. CL and Fra. MC. CA fractions exhibiting the highest activity (lowest IC_50_). Comprehensive metabolite profiling of the three extracts and their most active fractions using LC-QTOF-MS/MS revealed the presence of 99 metabolites, including organic and phenolic acids, catechins, flavonoids and bioflavonoids, lignans, diterpenes, and fatty acids. Among these, the highly abundant flavonoid rutin, epicatechin, the phenolic acid 3,5-di-*p*-coumaroylquinic acid, and the biflavonoid cupressuflavone were isolated from the CL and CA extracts and subsequently characterized by NMR and anti-lipase bioassay. Cupressuflavone exhibited the lowest IC_50_ value (3812 ± 450 µg/mL), making it approximately 3.89 times more potent against *A. baumannii* lipases compared to the positive control, orlistat. Additionally, multivariate data analysis (PLS) highlighted 25 key metabolites as the most discriminant for lipolytic activity inhibition across the studied samples. These included two phenolic acids, one catechin, three flavonoids, three biflavonoids, two lignans, six diterpene acids, and eight fatty acids. In conclusion, coniferous plants, especially *Cupressus* species, may represent a promising source of novel antivirulence agents against *A. baumannii* lipases. However, further research, including purification and in-depth in vitro, in vivo, and clinical studies, is recommended to fully evaluate the biological potential of the major identified compounds.

## Supplementary Information

Below is the link to the electronic supplementary material.

Supplementary Material 1

## Data Availability

The authors confirm that the data supporting the finding of this study are available within the article, its supplementary material and its ESI.

## Abbreviations

A. baumannii: *Acinetobacter baumannii*
%A: Percentage change in absorbance
A410: Absorbance at 410 nm
CA: *Cupressus arizonica Greene*
CL: *Cupressus lusitanica Mill*
DMSO: Dimethyl sulfoxide
EIC: Effective inhibitory concentration
ESI: Electrospray ionization
Fra.: Fraction
IC50: Half-maximal inhibitory concentration
LB: Luria–Bertani
LC-MS: liquid chromatography-mass spectrometry
LC-QTOF-MS/MS: Liquid chromatography-quadrupole time-of-flight tandem mass spectrometry
MC: Methylene chloride
Me: Methanol
MHB: Muller Hinton broth
MIC: Minimum inhibitory concentration
OD600: Optical density at 600 nm
PC: *Pinus canariensis C.Sm*
PLSPNP: Partial least square analysisp-nitrophenol
PNPP: p-nitrophenyl palmitate
SEM: Standard error of the mean
Sub-Fra.: Sub-fraction
Sub-MICTPF: Below minimum inhibitory concentration1, 3, 5- triphenyl formazan
TTC: 2, 3, 5- triphenyl tetrazolium chloride
WHO: World Health Organization
VIP: Variable importance in the projection
