# Supplementary material for: Targeting Acinetobacter baumannii lipase by coniferous species through metabolomics supported approach
Source: Sci Rep. 2025 Sep 23;15:32649. doi: 10.1038/s41598-025-16654-6 (PMC12457599; doi:10.1038/s41598-025-16654-6)
Supplement: Supplementary file 1 — Supplementary Material 1 [file 41598_2025_16654_MOESM1_ESM.docx]

**Supplementary material**

**Targeting *Acinetobacter baumannii* lipase by coniferous species through metabolomics supported approach**

Rania M. Kamal^1*^, Ali M. El-Halawany^1^, Mohamed S. Hifnawy^1^, Asmaa M. Otify^1^, Walaa G. Fahmy^2^, Noha M. Elhosseiny^2^, Ahmed S. Attia^2,3^, Basma M. Eltanany^4^, Laura Pont^5,6^, Fernando Benavente^5^, Inas Y. Younis^1**^, Manal M. Sabry^1**^

^1^ Department of Pharmacognosy, Faculty of Pharmacy, Cairo University, Cairo 11562, Egypt.

^2^ Department of Microbiology and Immunology, Faculty of Pharmacy, Cairo University, Cairo 11562, Egypt.

^3^ School of Pharmacy, Newgiza University, Giza 12588, Egypt.

^4^ Department of Pharmaceutical Analytical Chemistry, Faculty of Pharmacy, Cairo University, Cairo 11562, Egypt.

^5^ Department of Chemical Engineering and Analytical Chemistry, Institute for Research on Nutrition and Food Safety (INSA·UB), University of Barcelona, Barcelona 08028, Spain.

^6^ Serra Húnter Program, Generalitat de Catalunya, Barcelona 08007, Spain.

*Correspondence: [rania.ahmed@pharma.cu.edu.eg](mailto:rania.ahmed@pharma.cu.edu.eg) (R.M.K.)

** Both authors contributed equally.


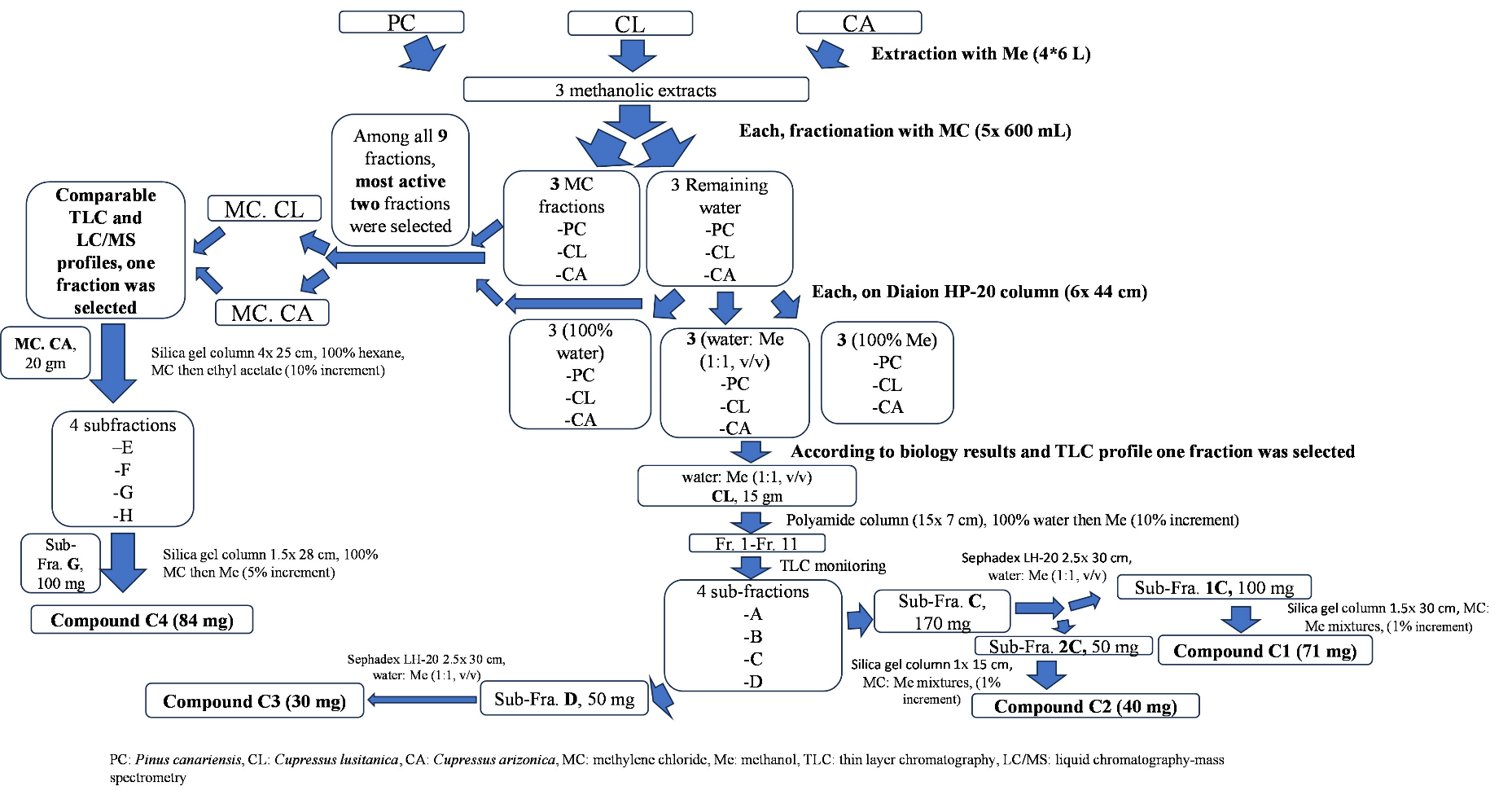


Figure S1: Diagram for extraction, fractionation and isolation of C1-C4 compounds

Quinic acid

*m/z* 191.0550 (C_7_ H_12_ O_6_)

**2**

[M−H]^−^

[M−H−18]^−^

[M−H−18−18]^−^

[M−H]^−^

[M−H−18]^−^

Shikimic acid

*m/z* 173.0434 (C_7_ H_10_ O_5_)

**4**

Figure S2: MS/MS spectra of metabolites 2 and 4 (Table 3)

[M−H]^−^

[M-H-44]^−^

Protocatechuic acid

*m/z* 153.0182 (C_7_ H_6_ O_4_)

**7 (negative ESI mode)**

Protocatechuic acid

*m/z* 155.0336 (C_7_ H_6_ O_4_)

[M+H]^+^

[M+H–44] ^+^

**7 (positive ESI mode)**

[M−H]^−^

[M−H−162]^−^

[M−H−162−44]^−^

Protocatechuic acid hexoside

*m/z* 315.0707 (C_13_H_16_O_9_)

**122**

Figure S3: MS/MS spectra of metabolites 7 and 12 (Table 3)

[M−H]^−^

[M−H−44]^−^

*p-*coumaric acid

*m/z* 163.0393 (C_9_H_8_O_3_)

**17 (negative ESI mode)**

[M+H]^+^

[M+H-44]^+^

*p-*coumaric acid

*m/z* 165.0393 *(*C_9_ H_8_O_3_)

**17 (positive ESI mode)**

Figure S4: MS/MS spectra of metabolite 17 (Table 3)

Catechin-7-O-*β*-D-glucopyranoside
 *m/z* 451.1221 (C_21_H_24_O_11_)

[M−H−162]^−^

[M−H]^−^

**24**

[M−H−288]^−^

[M−H−288−44]^−^

Procyanidin B
*m/z* 577.1332 (C_30_H_26_O_12_)

**25 (negative ESI mode)**

Procyanidin B
*m/z* 579.1527 (C_30_H_26_O_12_)

[M+H-288]^+^

[M+H]^+^

**25 (positive ESI mode)**

Figure S5: MS/MS spectra of metabolites 24 and 25 (Table 3)

Rutin
*m/z* 609.1437 (C_27_H_30_O_16_)

[M−H]^−^

[M−H−146]^−^

[M−H−146−162]^−^

**32 (negative ESI mode)**

Rutin
*m/z* 611.1615 (C_27_H_30_O_16_)

[M+H]^+^

**32 (positive ESI mode)**

[M+H−146−162]^−^

[M−H]^−^

[M−H−308]^−^

Kaempferol-3-O rutinoside
*m/z* 593.1489 (C_27_H_30_O_15_)

**36**

Figure S6: MS/MS spectra of metabolites 32 and 36 (Table 3)

[M−H]^−^

[M−H−162]^−^

Cupressuflavone
*m/z* 537.0814 (C_30_H_18_O_10_)

**53 (negative ESI mode)**

**A**

000000000000000000

[M+H]^+^

[M+H–162]^+^

**53 (positive ESI mode)**

Cupressuflavone
*m/z* 539.0812 (C_30_H_18_O_10_)

[M-H]^-^

Figure S7: MS/MS spectra of metabolite 53 (Table 3).

Lariciresinol hexoside
*m/z* 521.1995 (C_26_H_34_O_11_)

**60**

[M−H]^−^

[M−H−162]^−^

Figure S8: MS/MS spectrum of metabolite 60 (Table 3)

[M−H]^−^

[M−H−18−28]^−^

[M−H−18−28−18]^−^

Imbricatolic acid
*m/z* 321.2424 (C_20_H_34_O_3_)

**67**

Figure S9: MS/MS spectra of metabolite 67 (Table 3)

[M−H]^−^

[M−H−18]^−^

[M−H−18−18]^−^

Trihydroxy-octadecadienoic acid
*m/z* 327.2163(C_18_H_32_O_5_)

**72**

Figure S10: MS/MS spectrum of metabolite 72 (Table 3)

Figure S11: Chemical structures of the C1-C4 isolated compounds

**A**

**B**

Figure S12: PLS validation parameters: a. the permutation tests of lipolytic activity inhibition and b. PLS derived relationship between observed vs predicted lipolytic inhibition activity
